# Large-Scale Screening of Per- and Polyfluoroalkyl Substance Binding Interactions and Their Mixtures with Nuclear Receptors

**DOI:** 10.3390/ijms25158241

**Published:** 2024-07-28

**Authors:** Saptarshi Roy, James Moran, Keerthana Danasekaran, Kate O’Brien, Sivanesan Dakshanamurthy

**Affiliations:** 1 College of Humanities and Sciences, Virginia Commonwealth University, 907 Floyd Ave, Richmond, VA 23284, USA; 2 College of Arts & Sciences, Georgetown University, 3700 O St NW, Washington, DC 20057, USA; 3 College of Arts and Sciences, University of Rochester, 500 Joseph C. Wilson Blvd, Rochester, NY 14627, USA; 4 Davidson College, 405 N Main St, Davidson, NC 28035, USA; 5 Lombardi Comprehensive Cancer Center, Georgetown University, 3700 O St NW, Washington, DC 20057, USA

**Keywords:** nuclear receptor, PFAS, PFAS screening, molecular docking, orthosteric site, allosteric site

## Abstract

Despite their significant impact, comprehensive screenings and detailed analyses of per- and polyfluoroalkyl substance (PFAS) binding strengths at the orthosteric and allosteric sites of NRs are currently lacking. This study addresses this gap by focusing on the binding interaction analysis of both common and uncommon PFAS with the nuclear receptors (NRs) vitamin D receptor (VDR), peroxisome proliferator-activated receptor gamma (PPARγ), pregnane X receptor (PXR), and estrogen receptor alpha (ERα). Advanced docking simulations were used to screen 9507 PFAS chemicals at the orthosteric and allosteric sites of PPARγ, PXR, VDR, and ERα. All receptors exhibited strong binding interactions at the orthosteric and allosteric site with a significant number of PFAS. We verified the accuracy of the docking protocol through multiple docking controls and validations. A mixture modeling analysis indicates that PFAS can bind in various combinations with themselves and endogenous ligands simultaneously, to disrupt the endocrine system and cause carcinogenic responses. These findings reveal that PFAS can interfere with nuclear receptor activity by displacing endogenous or native ligands by binding to the orthosteric and allosteric sites. The purpose of this study is to explore the mechanisms through which PFAS exert their endocrine-disrupting effects, potentially leading to more targeted therapeutic strategies. Importantly, this study is the first to explore the binding of PFAS at allosteric sites and to model PFAS mixtures at nuclear receptors. Given the high concentration and persistence of PFAS in humans, this study further emphasizes the urgent need for further research into the carcinogenic mechanisms of PFAS and the development of therapeutic strategies that target nuclear receptors.

## 1. Introduction

PFAS, synthetic forever chemicals used for 60 years, are exposed to 99% of the human population every day. PFAS are integral to various consumer goods, providing waterproof, grease-proof, and nonstick properties [1]. Drinking water, in particular, has been identified as a major source of PFAS exposure to humans [2]. PFAS are increasingly recognized for their adverse health effects, including cancer, metabolic issues, and reproductive issues. Higher exposure to these chemicals has been linked to irregularities in menstrual cycles and increased cycle length [3]. The persistence and bioaccumulation of PFAS raise considerable health concerns, including cancer risk. The carcinogenic potential of PFAS is attributed to factors such as action–metabolism disruption, endocrine system interference, and epigenetic changes [4]. In addition to their carcinogenic potential, PFAS continue to harm humans through their impact on reproductive systems, developmental stages, and other organs, leading to kidney or liver disease [5]. Importantly, there is evidence of associations between PFAS exposure and kidney, testicular, ovarian, thyroid, and endometrial cancers [6,7,8,9,10,11,12].

The physicochemical properties of PFAS significantly influence their binding interactions with nuclear receptors. The hydrophobicity and stability of PFAS contribute to their persistence and potential bioaccumulation, further influencing their ability to interact with and disrupt the normal functioning of nuclear receptors. Such interactions can lead to significant biological effects, given the role of nuclear receptors in regulating gene expression related to development, metabolism, homeostasis, and cancer. Understanding these interactions is essential for assessing the toxicological impacts of PFAS on human health. PFAS consist of a carbon backbone attached to fluorines, which results in a nonpolar hydrophobic nature, similar to that of the endogenous ligands of nuclear receptors like VDR, PPARγ, PXR, and ERα. This nonpolarity allows the chemicals to bind to nonpolar ligand binding domains, composed of mainly nonpolar amino acids. Long and short PFAS chains have been shown to disrupt the function of nuclear receptors such as PXR, causing the overactivation of the receptor, and leading to endocrine disruption, oxidative stress, and hepatic steatosis [13]. It has been shown that PFOA competitively binds at the VDR [14]. When PFAS are bound to the VDR, the VDR cannot properly regulate genes that are involved in calcium and phosphate homeostasis, immune response, and cellular proliferation and differentiation [15]. PFOA, when bound to PPARγ, has shown anomalies in bone development and adipose tissue homeostasis [16]. PFAS compounds, such as PMOH and PMPP, have also been shown to be weak activators of PPARγ [17].

Previous studies showed correlations with a greater PFAS exposure and increased rate of thyroid cancer [18]. Colorectal cancer susceptibility increases when the VDR is dysregulated, a condition that can be induced by PFAS through their binding to the VDR, as well as to other nuclear receptors [19]. The VDR has been shown to regulate blood pressure, work against organ failure, and play as a factor for autoimmunity, angiogenesis, inflammation, and vascular cell activity [20]. Given the regulatory functions of the VDR, when the competitive binders of calcitriol like PFOA bind instead, structural flexibility is decreased, leading to an altered response of the vitamin D-responsive genes [21]. As more of the PFAS are able to bind the VDR, the function of 1,25-dihydroxyvitamin D, an agent that works against age-related osteoporosis, becomes limited, thus leading towards a higher likelihood of developing osteoporosis [22]. There are many common PFAS molecules that could trigger the effects of osteoporosis, like PFOA or PFOS as these are the most abundant in relation to humans [23].

The PXR is involved in the regulation of intermediate metabolism through the trans-activation and trans-repression of genes controlling glucose, lipid, cholesterol, bile acid, and bilirubin homeostasis [24]. PXR is composed of a DNA binding domain (DBD), involved in receptor dimerization and binding of specific DNA sequences; an H region; a flexible domain connecting the DBD with the ligand binding domain (LBD); and a C-terminal LBD [24]. PXR was also originally characterized as the key transcription factor that activates hepatic genes, encoding drug-metabolizing enzymes and drug efflux transporters, and protects the body from harmful foreign toxicants or endogenous toxic substances [24]. Studies have been conducted, detailing the interactions between PFAS and the human PXR receptor, with binding typically leading to an overactivation of the receptor [13]. The overactivation of PXR is linked with potential endocrine disruption, oxidative stress, hepatic steatosis, and other adverse drug interactions [13]. 

PPARγ plays a major role in cell differentiation, lipid metabolism, and glucose homeostasis [25]. Thiazolidinediones (TZDs) are agonists that bind to the PPARγ, which are prescribed for the treatment of type II diabetes. When associated with PPARγ, PFAS have been shown to have effects on adipose tissue and bone differentiation, which leads to significant cancerous effects [16]. Some adipose tissue-related effects like obesity have been associated with younger children that had a higher exposure to specific PFAS like PFOA and PFHxS [16]. Correlations have shown that with increased PFAS concentration, there is an increased count of adipose tissue, leading to a greater BMI and fat deposition.

ERα is a ligand-activated nuclear hormone receptor responsible for gene expression in the nucleus, by estradiol, which is the most prevalent during primary and secondary reproductive years. Estradiol diffuses into the cell and binds to ERα in the cytoplasm. ERα, composed of 595 amino acids, has six domains: A/B, C, D, and E/F. Estradiol binds to the LBD within domain E, forming hydrogen bonds with Glu353, Arg394, and His524, causing a conformational change that moves alpha helix 12 and opens the Activation Factor 2 (AF-2) site for coactivator recruitment and ERα dimerization. PFAS have been shown to bind to human ERα at greater rates, leading to an increased activation of the estrogen receptor and overexpression of estrogen [26]. The AF-1 and AF-2 sites are key regulators of transcriptional activity and are hormonally activated. The orthosteric and allosteric sites containing ERα LBD and AF-2 sites were used for PFAS docking simulations.

Given the hazardous effects and ability to disrupt NR functionality, there is significant interest in understanding the binding interactions between PFAS and NRs. In an attempt to help explore the mechanisms by which PFAS bind and disrupt NR activation, a unique screening study was performed that investigates over 9000 PFAS chemical interactions with VDR, PPARγ, PXR, and ERα, at both their orthosteric and allosteric binding sites. The novelty of this study is highlighted by the inclusion of PFAS binding interactions at allosteric binding sites. The findings demonstrated a large range of PFAS that had an ability to bind to the VDR, PPARγ, PXR, and ERα, with binding affinities comparable to or greater than those of endogenous ligands, at orthosteric and allosteric sites.

## 2. Results and Discussion

### 2.1. Docking Verification: Cross-Docking of Known Ligands and Non-Native Ligands and Comparison with Experimental Binding Affinity

The interactions of PFAS, native ligands, and non-native ligands to the VDR, PXR, PPARγ, and ERα were examined. This study’s approach involved validating the Autodock Vina docking protocol by docking known ligands and confirming the accuracy of native ligands by cross-docking of non-native ligands for each nuclear receptor. These steps are important to ensure the reliability of the docking protocol. The docking results highlighted below demonstrate that native ligands are docked correctly to their respective nuclear receptors, as seen by low RMSD values from the X-ray binding ligand conformation, highlighting the precision of Vina in combination with the docking protocol. The examination of non-native ligand docking results further validates the docking protocol.

#### 2.1.1. Cross-Docking of Known Ligands: VDR

**Figure 1:**
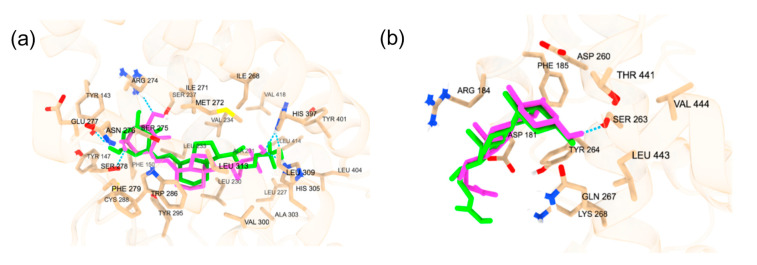
(**a**) The redocked calcitriol (pink) and native co-crystalized structure of calcitriol (green) (PDB ID: 1IE9); The blue dotted lines are the hydrogen bonds. (**b**) The redocked lithocholic acid (pink) and native co-crystalline structure of lithocholic acid (green) are displayed (PDB ID: 4Q0A). The blue dotted lines are the hydrogen bonds.

The accuracy of Autodock Vina predictions was verified using cross-docking of native ligands at the orthosteric and allosteric sites of the VDR (Figure 1). Two native ligands were docked to verify the molecular docking procedure. The docking of the native ligands, calcitriol (PDB ID: 1IE9, resolution: 1.40 Å) and lithocholic acid (PDB ID: 4Q0A, resolution: 1.90 Å), was compared to the co-crystalline structures. The superimposed structures for calcitriol and lithocholic acid are shown in Figure 1. The docking at both the orthosteric and allosteric sites yielded similar accuracies, with calcitriol displaying an RMSD of 1.31 Å and lithocholic acid exhibiting an RMSD of 1.306 Å. The amino acid residues within 5 Å of the orthosteric site include TYR 143, TYR 147, PHE 150, SER 237, LEU 233, ARG 274, CYS 288, GLU 277, SER 278, SER 275, TYR 295, ILE 271, TRP 286, ASN 276, PHE 279, LEU 313, MET 272, VAL 300, LEU 309, HIS 305, HIS 397, ILE 268, ALA 303, LEU 404, TYR 401, LEU 230, VAL 234, ALA 231, LEU 227, VAL 418, and LEU 414. In the co-crystalline structure, calcitriol forms hydrogen bonds with HIS 397 and TYR 143, and in the redocked structure, it forms additional hydrogen bonds with ARG 274, SER 278, and HIS 305. The amino acid residues surrounding the allosteric site within 5 Å are ARG 184, ASP 181, ASP 260, TYR 264, SER 263, LYS 268, LEU 443, PHE 185, THR 441, VAL 444, and GLN 267. Although the co-crystalline structure of lithocholic acid did not show hydrogen bonding with the VDR, the redocked ligand formed a hydrogen bond with SER 263, demonstrating the flexibility of the structure and its capacity to optimize binding with VDR. The docking score for calcitriol is −12.3 kcal/mol, indicating strong interactions with the VDR LBD, whereas the docking score for lithocholic acid is −6.6 kcal/mol, suggesting much weaker interactions.

#### 2.1.2. Cross-Docking of Known Ligands: PPARγ

**Figure 2:**
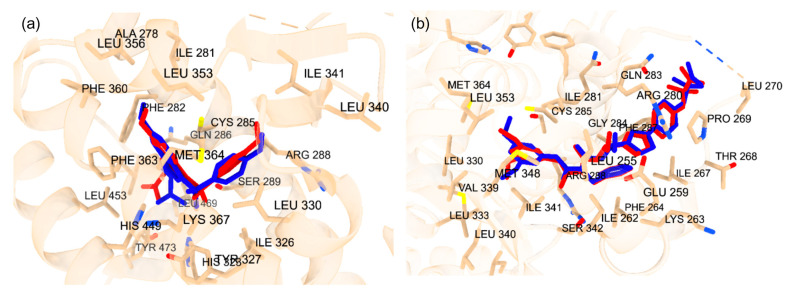
(**a**) Superimposed co-crystal structure in blue (PDB ID: 3ET3) and docked binding pose of ET1 in red. Amino acid residues within 5 Å of co-crystal structure are shown in stick form. (**b**) Superimposed co-crystal structure in blue (PDB ID: 5GTO) and docked binding pose of T35 in red. Amino acid residues within 5 Å of co-crystal structure are shown in stick form.

To confirm the accuracy of the docking protocol for the PPARγ orthosteric binding site, the binding pose of the native ligand, ET1, was analyzed. The pose obtained via molecular docking was compared with the co-crystallized structure of ET1 (PDB ID: 3ET3), resulting in an RMSD of 1.017 Å (Figure 2a). Further, the docked ET1 at the orthosteric site formed a hydrogen bond with T473. The docking protocol was similarly validated for the allosteric binding site. The pose of the PPARγ allosteric site native ligand, T35, was compared with the co-crystallized structure of T35 (PDB ID: 5GTO), yielding an RMSD of 0.758 Å (Figure 2b). Additionally, the docked T35 formed hydrogen bonds with residues L270 and Q282. These findings validate the molecular docking protocol efficacy in accurately placing ligands within their respective binding pockets in the correct orientation, confirming that the parameters for docking small molecules to the LBD of PPARγ successfully reproduce the experimentally observed binding poses of known ligands.

#### 2.1.3. Cross-Docking of Known Ligands: PXR

**Figure 3:**
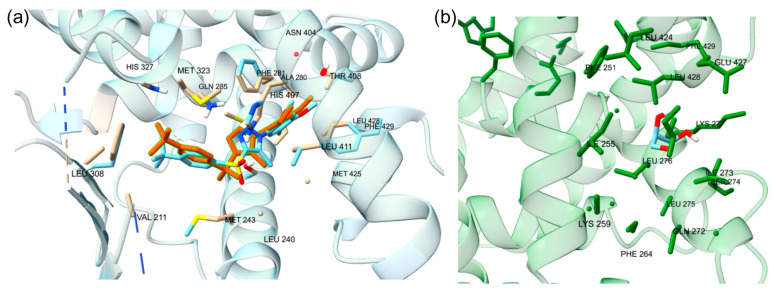
(**a**) Superimposed conformation of redocked 4WH ligand onto co-crystallized 4WH ligand on PXR protein. The orange ligand represents the redocked ligand. Amino acid residues within distance of 5 Å are labeled. (**b**) Superimposed conformation of redocked glycerol ligand onto co-crystallized allosteric site on PXR protein. Light blue ligand represents redocked ligand. Amino acid residues within distance of 5 Å are labeled.

To confirm the docking protocol for the orthosteric site of PXR, the native ligand 4WH was redocked onto the orthosteric site of the PXR. This approach was used to validate the accuracy of the docking protocol by comparing the computationally predicted binding mode to the experimentally determined position and orientation of the ligand in the PXR. Figure 3a displays the superimposed conformation of the redocked ligand and the co-crystallized ligand on the PXR. The RMSD value was calculated for the superimposition, resulting in a value of 0.793 Å. This value falls within the RMSD criterion of less than 2 Å, indicating a high level of docking accuracy. To verify the docking protocol for the allosteric AF-2 site of PXR, glycerol was redocked onto the allosteric site of the PXR. Figure 3b shows the superimposed conformation of the redocked ligand and the co-crystallized ligand on the PXR. The RMSD value was calculated for the superimposition, resulting in a value of 0.098 Å. This value falls within the RMSD criterion of less than 2 Å, indicating a high level of docking accuracy. 

#### 2.1.4. Cross-Docking of Known Ligands: ERα

**Figure 4:**
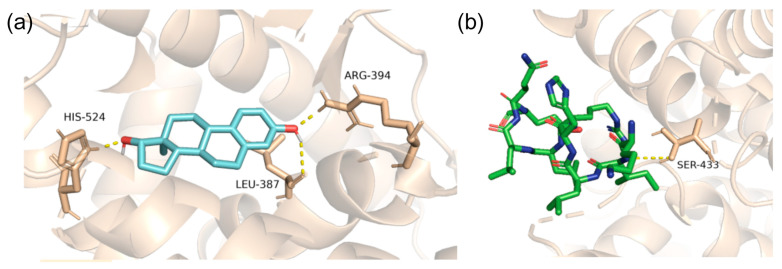
(**a**) The redocked estradiol is displayed at the orthosteric site. (**b**) The redocked SRC-1 is displayed at the allosteric site. The yellow dotted lines are the hydrogen bonds. The amino acid residues involved with hydrogen bonding are displayed.

To validate the docking protocol, endogenous ligands were redocked at the orthosteric and allosteric site of the ERα, to produce binding conformations. Estradiol on the 1ERE protein–ligand complex is depicted in Figure 4. The analysis revealed a docking score of −11.1 kcal/mol for estradiol. Further, the arrangement of the docked ligand closely superimposed the native ligand of the 1ERE complex structure, providing strong validation for the docking methodology. At the AF-2 site, the same protocol for steroid receptor coactivator-1 (SRC-1) was followed to show accuracy for docking at the allosteric site. Through computational docking, the binding of SRC-1 to specific residues, particularly SER-433, was noticed. The calculations yielded a binding affinity of −9.9 kcal/mol for this interaction, displaying an accurate docking protocol, and the redocked ligand is displayed in Figure 4. The protocol for docking is deemed to be accurate for both protein files for the ERα due to the redocked ligands superimposing the native ligands from the protein files.

#### 2.1.5. Cross-Docking of Non-Native Ligands: VDR

To test the accuracy of Autodock Vina’s results, other proteins’ endogenous ligands were docked, like chenodeoxycholic, estradiol, Tretinoin, 15-deoxy-Δ-12,14-Prostaglandin, Triiodothyronine, onto the orthosteric and allosteric site of the VDR. The results supported Autodock Vina’s accuracy as it held calcitriol as the greatest docking score among other native ligands for other proteins in their respective orthosteric site, as shown in Table 1. At the orthosteric site, calcitriol had a docking score of −12.3 kcal/mol whereas the highest docking score for the crossed docked ligands was −9.4 kcal/mol by chenodeoxycholic and estradiol. As depicted in Table 1, Autodock Vina’s accuracy has been further established as the same molecules were docked in the allosteric site of the VDR, and still lithocholic acid had the greatest docking score. In comparison to the crossed docked ligands, LCA does not have a significantly greater docking score compared to other ligands. 

#### 2.1.6. Cross-Docking of Non-Native Ligands: PPARγ

In addition to verifying the protocol’s binding pose accuracy, the docking scores’ accuracy was also verified. This was performed by docking the native ligands of other nuclear receptors to the orthosteric PPARγ binding site and comparing the obtained docking scores to known native and synthetic ligands of PPARγ. In total, one native and one synthetic ligand of PPARγ, as well as six non-endogenous ligands, were docked into the LBD of PPARγ (PDB ID: 3ET3) (Table S1). The protocol gave the two highest docking scores to the known ligands while the six ligands not endogenous to PPARγ were given lower docking scores. These results thus confirm that the parameters for obtaining the docking scores are accurate and can be relayed to the docking of PFAS as depicted in Table 2. All ligands are bound to the orthosteric binding site and are within 5 Å of known amino acid residues including HIS323, TYR327, and LEU330.

#### 2.1.7. Cross-Docking of Non-Native Ligands: PXR

Docking experiments with various native ligands of PXR were performed on the orthosteric and allosteric AF-2 site to evaluate the accuracy of the docking protocol. The binding affinities of these native ligands were compared with those of external receptor ligands (Table S2). Vitamin K2 displayed the highest binding affinity of −9.7 kcal/mol among PXR native ligands at the orthosteric LBD site and Lovastatin displayed the highest binding affinity of −5.6 kcal/mol at the allosteric AF-2 site. Importantly, all of PXR’s native ligands exhibited superior binding affinities to the orthosteric site compared to the native ligands collected from external receptors, with the exception of PXR’s native ligand Schisandrin A. However, literature studies have shown that this compound activates human PXR with a similar efficacy and potency as rifampicin, which is known to be a relatively weak PXR binder [27,28]. Similarly, at the AF-2 site, most of PXR’s native ligands displayed higher binding affinities to the allosteric binding site compared to the native ligands collected from external receptors, with the exception of 5-beta-pregnane-3-20-dione and 3-keto-lithocholic acid. However, these ligands had binding affinity values of −4.6 kcal/mol, relatively close to the native ligand with the highest binding affinity that belonged to an external receptor, which was −4.7 kcal/mol. This minor difference could stem from the larger size and intricacy of the former ligands, making their precise docking at the allosteric site more challenging. Given the remarkably close resemblance of these outlier values to the optimal binding affinity of an external receptor’s native ligand, the docking methodology employed evidently retains its accuracy.

#### 2.1.8. Verification of Experimental Binding Affinity Values for Docking Accuracy

To further validate the accuracy of the docking protocol for the orthosteric site, the IC_50_, EC_50_, Ki, and Kd values of the native ligands were collected. The 4WH reference ligand’s EC_50_ value of 880 nM was chosen as the primary experimental data point because it surpassed the IC_50_ value of 340 nM for the reference ligand. Further, this EC_50_ binding affinity was translated into a value of −8.3 kcal/mol, which closely matched the binding affinity of the redocked 4WH ligand in its most similar conformation to the co-crystallized ligand within the PXR protein. For the VDR, the Kd value for calcitriol is 0.06 nM, while the Kd value for lithocholic acid is 330 nM. At the PPARγ, T35 had an EC_50_ value of 183 nM. In the ERα, estradiol had a max Ki value of 100nM, max Kd value of 100nM, max IC_50_ value of 46 nM, and max EC_50_ value of 10 nM. The experimental values of the glycerol, ET1, and SRC-1 reference ligands were unable to be identified. Glycerol plays a function when binding to the allosteric site of the PXR, while ET1 binds to the orthosteric site of the PPARγ, and SRC-1 interacts with the allosteric site of the ERα.

### 2.2. Virtual Screening of PFAS Molecules against VDR, PXR, PPARγ, and ERα

A total of 9903 PFAS were processed onto the VDR, PXR, PPARγ, and ERα. Of the 9903 chemicals, 396 ligands were excluded from this study as Vina was not able to dock them onto the protein. A total of 9507 ligands were completely processed and docked onto the orthosteric and allosteric site of the VDR, PXR, PPARγ, and ERα. There was similarity in structure within the top PFAS among the individual receptor binding sites. For example, the majority of the top PFAS at the VDR orthosteric site displayed many similarities in their structures. However, top PFAS at differing NRs had differences in structure due to the nonidentical amino acid residues occupying the binding sites. In terms of PFAS structure, fluorinated carbon rings and chains were common in the structure of various PFAS, with some also incorporating elements such as oxygen, nitrogen, hydrogen, and sulfur, like sulfonic acid. Despite these variations, the PFAS displayed structural likeness. Examining the structural similarities and differences among prominent PFAS bindings to NR is key for identifying the properties that contribute to high binding energy and NR activation.

#### 2.2.1. PFAS Virtual Screening: VDR Orthosteric Site

**Figure 5:**
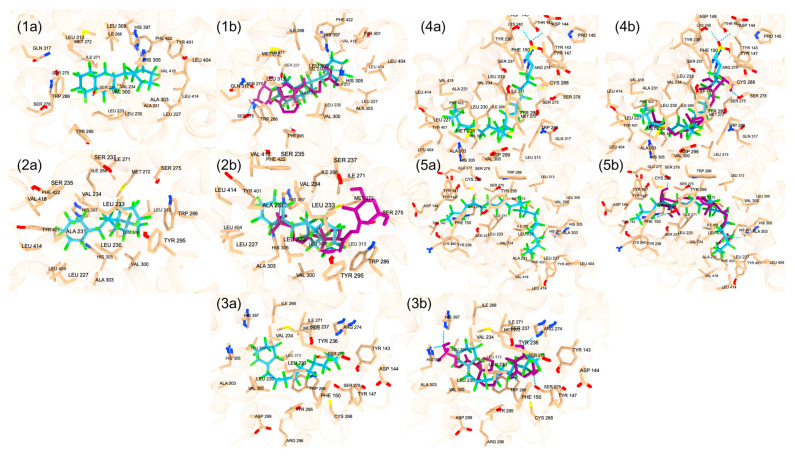
(**1a**) The top PFAS chemical (blue) ranked by the docking score at the orthosteric site is displayed, Perfluoroperhydrobenzyl tetralin, under the classification of Polycyclic Perfluoroalkane. (**1b**) Perfluoroperhydrobenzyl tetralin and the redocked calcitriol (purple) are displayed. (**2a**) The second top PFAS chemical (blue) by docking score at the orthosteric site is displayed, DTXSID60881337, under the classification of Polycyclic Perfluoroalkane. (**2b**) DTXSID60881337 and the redocked calcitriol (purple) are displayed. (**3a**) The third top PFAS chemical (blue) by docking score at the orthosteric site is displayed, DTXSID101023399, under the classification of Cyclic Perfluoroalkane. (**3b**) DTXSID101023399 and the redocked calcitriol (purple) are displayed. (**4a**) The fourth top PFAS chemical (blue) by docking score at the orthosteric site is displayed, DTXSID701036930, under the classification of Perfluoroalkyl Sulfonic Acids. (**4b**) DTXSID701036930 and the redocked calcitriol (purple) are displayed. (**5a**) The fourth top PFAS chemical (blue) by docking score at the orthosteric site is displayed, Hexatetracontafluorodocosane, under the classification of Perfluoroalkane. (**5b**) Hexatetracontafluorodocosane and the redocked calcitriol (purple) are displayed. The hydrogen bonds are displayed.

There were 130 PFAS that bound to the orthosteric site of the VDR at a greater docking score than calcitriol (Table S3). The range of PFAS docking scores with greater binding affinity than calcitriol was from −12.4 kcal/mol to −14.7 kcal/mol, as the top 15 PFAS are shown in Table 3. Additionally, at the orthosteric site, there are 21 PFAS that had similar docking scores to calcitriol, all with a docking score of −12.3 kcal/mol. Among the remaining PFAS, DTXSID101034031 stood out the most, as it had a docking score of +2.5 kcal/mol at the orthosteric site, which was the only positive binder among the 9356 PFAS that had a lower docking score than calcitriol. The positive binding energy observed is due to PFAS being very large, with a molecular weight of 2290.668 g/mol, and it does not properly fit into the VDR (Figure S1). The inability of these large PFAS molecules to adequately accommodate within the binding site of the VDR could result in suboptimal interactions, leading to less favorable binding energy (Figure S1). The docking of the top five PFAS in comparison to the orthosteric endogenous ligand, calcitriol, is displayed in Figure 5. Among the top five PFAS, four only contained carbons and fluorines, and the fifth one had a sulfonic acid, but was still mainly composed of fluorines attached to a carbon chain (Figure S2). PFAS that had similar binding energy to calcitriol were different from the top ligands in the sense that they also contained other atoms that are not as commonly found in the top PFAS, such as nitrogen, iodine, more oxygen, sulfur, phosphate, and sulfonic acid. A major similarity between the top five PFAS binding ligands is the nonpolarity, as there are carbon fluorine bonds decorated throughout the molecule, negating the dipole–dipole charge and neutralizing it. DTXSID40881335 (Fluoropolymer—FP), DTXSID60881337 (Fluoropolymer—FP), and DTXSID101023399 (Fluoropolymer—FP) all contain rings with fluorines attached around it, causing structures that are nonpolar and stable enough to interact with the VDR, therefore giving a greater binding affinity. DTXSID701036930 (Perfluoroalkyl Sulfonic Acid—PFSA) and Hexatetracontafluorodocosane (Fluoropolymer—FP) have more linear-type structures, allowing for more stereochemistry about the structures, but their main nonpolarity gives a greater binding affinity when docked into the orthosteric site. These results demonstrate the strong binding that certain PFAS have at the orthosteric site of VDR. A total of 130 PFAS demonstrated greater binding affinity than calcitriol, highlighting the risks that PFAS may pose to the functionality of VDR.

#### 2.2.2. PFAS Virtual Screening: VDR Allosteric Site

**Figure 6:**
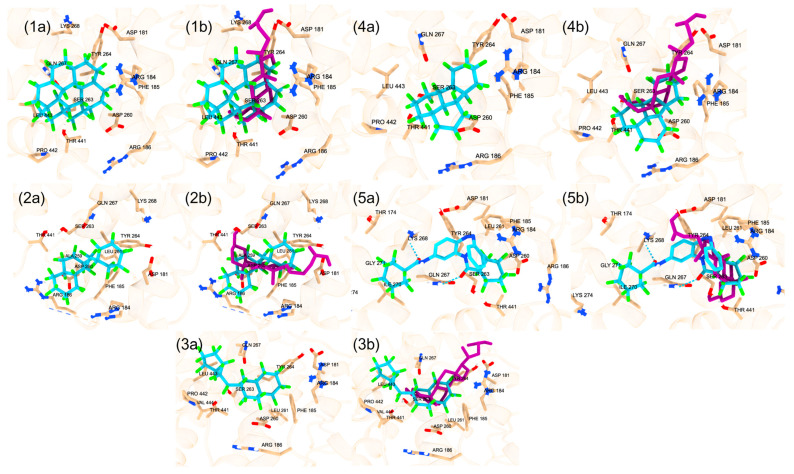
(**1a**) The top PFAS chemical (blue) by docking score at the allosteric site is displayed, DTXSID70597457, under the classification of Polycyclic Perfluoroalkane. (**1b**) DTXSID70597457 and lithocholic acid (purple) are displayed. (**2a**) The second top PFAS chemical (blue) by docking score at the allosteric site is displayed, DTXSID60881337, under the classification of Polycyclic Perfluoroalkane. (**2b**) DTXSID60881337 and lithocholic acid (purple) are displayed. (**3a**) The third top PFAS chemical (blue) by docking score at the allosteric site is displayed, Perfluoroperhydrobenzyl tetralin, under the classification of Polycyclic Perfluoroalkane. (**3b**) Perfluoroperhydrobenzyl tetralin and lithocholic acid (purple) are displayed. (**4a**) The fourth top PFAS chemical (blue) by docking score at the allosteric site is displayed, DTXSID90984683, under the classification of Polycyclic Perfluoroalkane. (**4b**) DTXSID90984683 and lithocholic acid (purple) are displayed. (**5a**) The fifth top PFAS chemical (blue) by docking score at the allosteric site is DTXSID501041016 under the classification of Polycyclic Perfluoroalkane. (**5b**) DTXSID501041016 and lithocholic acid (purple) are displayed. The hydrogen bonds are displayed.

A total of 2229 PFAS chemicals bound to the allosteric site of the VDR with greater binding affinity than lithocholic acid (Table S4). The scores of those PFAS ranged from −6.7 kcal/mol to −10.4 kcal/mol as shown in Table 4. A total of 412 PFAS had a similar docking score to that of lithocholic acid, with a docking score of −6.6 kcal/mol. Figure 6 shows the top five PFAS binding to the allosteric site of the VDR adjacent to the native ligand, lithocholic acid. The top five allosteric binding PFAS by docking score are displayed. Four of the five top PFAS have only carbons and fluorines, while one contains some oxygens, nitrogens, and hydrogen (Figure S3). PFAS that had similar binding energy to lithocholic acid did not have much structural similarities to LCA other than consisting of carbon chains and rings. DTXSID70597457 (Fluoropolymer—FP), DTXSID60881337 (Fluoropolymer—FP), DTXSID40881335 (Fluoropolymer—FP), DTXSID90984683 (Fluoropolymer—FP), and DTXSID501041016 (Perfluoroalkyl Acids—PFAAs) consist of carbon rings with fluorines attached. Similar to the orthosteric site, the allosteric site prefers nonpolar molecules. These results emphasize the importance of studying PFAS at the allosteric site of VDR. Over 2000 PFAS displayed strong binding with the allosteric site, demonstrating the potential risks PFAS may pose to the functionality of VDR.

#### 2.2.3. PFAS Virtual Screening: PPARγ Orthosteric Site

**Figure 7:**
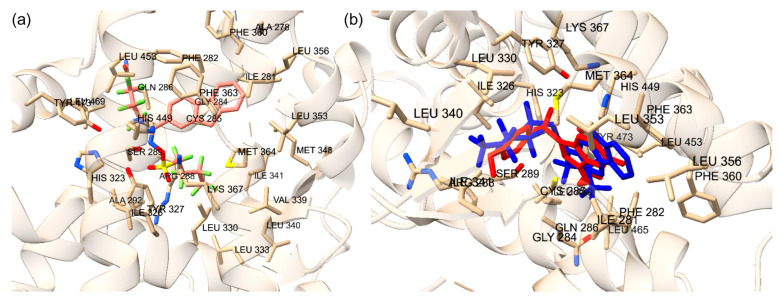
(**a**) The co-crystal structure of PPARy (PBD ID: 3ET3) and docked binding pose of the PFAS compound (DTXSID201033555) in the orthosteric binding site with the second-lowest docking score (−12.1). Amino acid residues within 5 Å of the co-crystal structure are shown in stick form. (**b**) The superimposed co-crystal structure of PPARy with ET1 in red (PDB ID: 3ET3) and the docked binding pose of the PFAS compound (DTXSID201033555) in blue bound to the orthosteric binding site with a docking score of −12.1. Amino acid residues within 5 Å of the co-crystal structure are shown in stick form.

**Figure 8:**
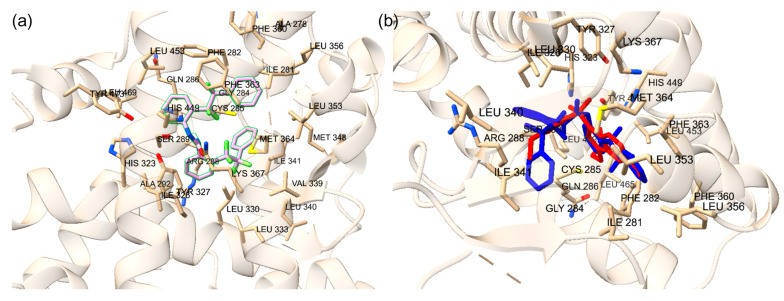
(**a**) The co-crystal structure of PPARy (PDB ID: 3ET3) and docked binding pose of the top PFAS compound (DTXSID20816403) in the orthosteric binding site with a docking score of −12.2. Amino acid residues within 5 Å of the co-crystal structure are shown in stick form. (**b**) The superimposed co-crystal structure of PPARy with ET1 in red (PDB ID: 3ET3) and the docked binding pose of the PFAS compound (DTXSID20816403) in blue bound to the orthosteric binding site with a docking score of −12.2. Amino acid residues within 5 Å of the co-crystal structure are shown in stick form.

For the orthosteric binding site, PFAS chemicals with a docking score greater than −9 kcal/mol were shortlisted given that the minimum docking score for a known ligand was −9 kcal/mol. Of the 9505 docked PFAS compounds into the orthosteric site, 1823 molecules were shown to have a binding affinity stronger than the endogenous ligand, ET1 (Table S5). The top 15 PFAS with the greatest docking scores were compiled in Table 2. The top 15 PFAS were bound to the PPARγ orthosteric site with binding affinities ranging from −11.3 to −12.2 kcal/mol, displaying the top-ranked PFAS in Figure 7 and second top-ranked in Figure 8. The top-ranked orthosteric PFAS had many structural similarities that were slightly different than that of the allosteric site of the PPARγ. The orthosteric PFAS included more functional groups that made the overall compound more polar. Some compounds contained stronger electron withdrawing groups like sulfonic acid and carbonyls, some with a mix of different functional groups, whereas one of the PFAS had no functional groups, resulting in no polarity. Therefore, the inference can be drawn that the structure of the orthosteric site has more polarity than the structure of the allosteric site of PPARγ, which explains why there are more functional groups, causing polarity, present in PFAS that bind at high affinities at the orthosteric site in comparison to that at the allosteric site. These results highlight the potential risks that PFAS may pose. Over 1800 PFAS have been identified to bind strongly to the orthosteric site of PPARγ, demonstrating the potentially negative impact PFAS may have on the functionality of PPARγ.

#### 2.2.4. PFAS Virtual Screening: PPARγ Allosteric Site

**Figure 9:**
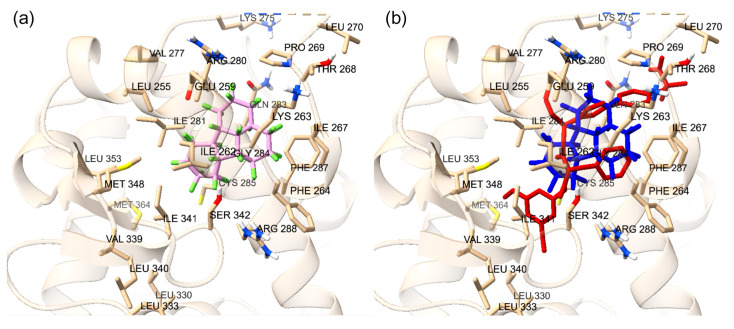
(**a**) The co-crystal structure of PPARy (PDB ID: 5GTO) and docked binding pose of the PFAS compound (DTXSID90984683) in the alternative binding site with the lowest docking score (−13.5). Amino acid residues within 5 Å of the co-crystal structure are shown in stick form. (**b**) The superimposed co-crystal structure of PPARy with T35 in red (PDB ID: 5GTO) and the docked binding pose of the PFAS compound (DTXSID90984683) in blue bound to the alternative binding site with a docking score of −13.5. Amino acid residues within 5 Å of the co-crystal structure are shown in stick form.

For the allosteric binding site, PFAS compounds with a docking score greater than −10 kcal/mol were shortlisted given that the minimum docking score for a known ligand into the allosteric binding site was −10 kcal/mol. Of the 9505 docked PFAS compounds, 693 compounds have a binding affinity greater than −10 kcal/mol, which are stronger binders than the endogenous ligand, T35 (Table S6). The structure and binding interactions of the top PFAS compounds were then analyzed, and the top PFAS at the allosteric site is displayed in Figure 9. These top PFAS molecules formed polar interactions with the following amino acid residues: HIS323, TYR473, SER289, CYS285, and ARG288. Previous research indicates that HIS323, TYR473, and SER289 form H bonds with known PPARγ agonists and are crucial for PPAR activation [29]. The top 15 PFAS have many similarities, as they all contain some form of cyclic features like rings and aromatic rings. The majority of the top 15 PFAS binding ligands have nonpolar substituents; however, there are some with ethers, esters, alcohol, and amine groups. A total of 693 PFAS displayed strong binding interactions with the allosteric binding site of PPARγ, demonstrating the significant risks PFAS may pose in the dysregulation of PPARγ.

#### 2.2.5. PFAS Virtual Screening: PXR Orthosteric Site

**Figure 10:**
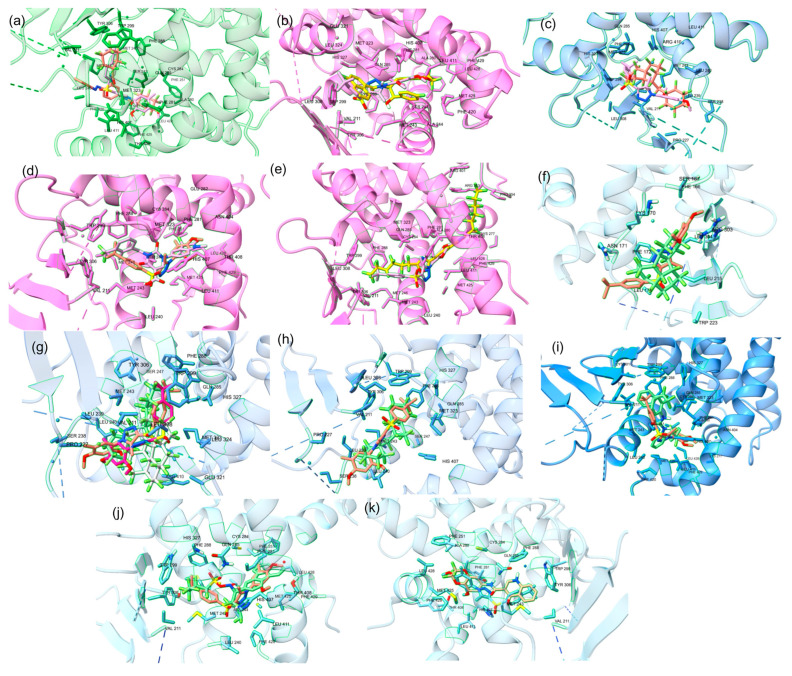
The 11 highest-binding-affinity PFAS superimposed conformations at the orthosteric site of the PXR are displayed along with the reference ligand, 4WH. (**a**) DTXSID501041016 (salmon) with a docking score of −13.5. (**b**) DTXSID401026885 (yellow) with a docking score of −13.1. (**c**) DTXSID60881337 (salmon) with a docking score of −12.6. (**d**) DTXSID701026989 (salmon) with a docking score of −12.4. (**e**) DTXSID70896735 (yellow) with a docking score of −12.1. (**f**) DTXSID10896198 (green) with a docking score of −12.1. (**g**) DTXSID90984683 (salmon)with a docking score of −12. (**h**) DTXSID70597457 (green) with a docking score of −12. (**i**) DTXSID801026875 (green) with a docking score of −12. (**j**) DTXSID60896264 (green)with a docking score of −12. (**k**) DTXSID40881335 (beige) with a docking score of −12. Amino acid residues within a distance of 5 Å are labeled.

The docking outcomes of PFAS molecules onto the orthosteric site of PXR were organized based on their descending order of binding affinities. Among these, 650 PFAS molecules exhibited binding affinities surpassing that of the reference ligand 4WH, which was −10.2 kcal/mol. The top ligands demonstrate the highest binding affinities shown in Table 5. Additionally, the superimposed conformations of the top ligands with the highest binding affinities are shown in Figure 10. These molecules share several structural similarities that may have contributed to their notable elevation in docking. At the orthosteric site, top-ranked PFAS chemicals 1, 2, 4, 5, 9, 10, and 11, namely DTXSID501041016, DTXSID401026885, DTXSID701026989, DTXSID70896735, DTXSID801026875, DTXSID60896264, and DTXSID40881335, exhibit indications of polycyclic aromaticity, exhibiting their ability for intramolecular resonance engagement as described below (Table S7). Upon the analysis, π-stacking interactions were noticed involving aromatic rings and play a crucial role, significantly contributing to the strength of ligand binding [30]. These interactions, characterized by traits like planarity and a discernible π-electron cloud above and below the rings, provide diverse interaction possibilities [30]. PFAS molecular binding (Figure 10 and Table 5) such as π π interactions and cation π, amide π, halogen π, and hydrogen bond interactions facilitated by heteroatoms are encompassed by these interactions [30]. It has been established that π π stacking is essential for favorable electron correlation [31]. The capacity of PFAS ligands to display resonance characteristics and distribute electrons uniformly across the entire molecule is crucial for strong binding affinities, as PFAS ligand–receptor interactions are influenced by the extent of electron cloud overlap. These findings emphasize the importance of aromatic π-stacking in influencing PFAS ligand–receptor interactions, providing valuable insights into the factors that contribute to strong binding affinities.

The PFAS fluorine atom’s unique characteristics enhance interactions between molecules. Its electronegativity forms strong bonds with the receptor, optimizing binding and stabilizing the ligand in the binding site. Further, the small size of fluorine atoms reduces clashes and increases PFAS ligand flexibility, leading to a more favorable alignment with the receptor, resulting in improved docking scores and potentially higher binding affinities. The significance of fluorine atoms in establishing high binding affinities between proteins and ligands finds empirical support in the existing literature. This can be supported by the binding affinity of PFAS to PXR (Figure 10 and Table 5). Notably, the analysis revealed that among the top 14 PFAS exhibiting the strongest binding affinities to the PXR receptor, fluorine substituents were attached to all carbon atoms constituting the ligand’s backbone, excluding the carbon atom present in the carboxylic acid group [13]. Of particular interest, a PFAS known for its substantial binding affinity, namely FTCA (5:3 fluorotelomer carboxylic acid), featured a carboxylic acid functional group [13]. This specific ligand shared a similar chemical structure with the PFAS denoted as DTXSID701026989 (Perfluoroalkyl Acids—PFAAs). This ligand demonstrated a distinctly high binding affinity to the orthosteric site within the PXR receptor. Further, all of the top-ranked PFAS that were docked at the orthosteric site of PXR had at least one fluorine substituent present, seeing a trend with ligands of higher binding affinities containing more fluorine substituents. A total of 650 PFAS displayed strong binding interactions with the allosteric binding site of PXR, demonstrating the significant risks PFAS may pose in the dysregulation of PXR.

#### 2.2.6. PFAS Virtual Screening: PXR Allosteric Site

The docking outcomes of PFAS molecules onto the allosteric AF-2 site of PXR were rank-ordered based on their descending order of binding affinities. Among these, 9148 PFAS molecules exhibited binding affinities surpassing that of the reference ligand glycerol, which was −2.3 kcal/mol. The top binding ligands had affinities ranging from −7.6 kcal/mol to −9.3 kcal/mol, and the PFAS ligand with the highest binding affinity to the allosteric AF-2 site of the PXR molecule was cobalt (Table S8). Many similarities in regards to the structure of these molecules could be seen when analyzing the binding affinities, with the primary ones being the presence of fluorine substituents and cyclic structures in 10 and 11, namely DTXSID40204383 and DTXSID301027559, of the top 13 binding ligands, respectively. As stated earlier, fluorine’s distinct properties amplify molecular interactions by creating robust bonds with PXR, enhancing binding of the PFAS ligand within the site. Additionally, the small size of fluorine atoms minimizes collisions and boosts ligand flexibility, promoting a stable cooperative alignment with the receptor. This alignment potentially elevates docking scores and enhances binding affinities. The existing literature highlighted the crucial role of fluorine atoms contributing to strong binding affinities between proteins and ligands in PXR orthosteric sites, but minimal research has been performed on the effects of fluorine on the allosteric site of PXR. However, for the purpose of this study, the effects are inferred to be rather similar. Further, the presence of cyclic structures has a significant effect on the ability of PFAS ligands to bind to the AF-2 allosteric site of PXR, as the AF-2′s ligand-dependent groove is made up of helices 3, 4, 5, and 12, which are primarily hydrophobic regions [28]. The cyclic structures found in PFAS ligands are made up of adjacent cyclohexane molecules that are completely surrounded by fluorine molecules whose electronegativities oppose each other, resulting in an overall nonpolar state of the PFAS ligands. Because the top ligands are nonpolar, they are more likely to exhibit a strong attraction to the AF-2 site due to the hydrophobic interaction between the ligand and receptor. A total of 9148 PFAS showed stronger binding interactions than glycerol at the allosteric binding site of PXR, demonstrating the risks PFAS may pose in the dysregulation of PXR.

#### 2.2.7. PFAS Virtual Screening: ERα Orthosteric Site

At the ERα, only 40 PFAS bind at a greater binding affinity than the endogenous ligand estradiol, ranging from −11.2 to −14.8 (Table S9). Among the top 10 PFAS ligands, there were considerable structural differences, as some displayed more linear characteristics, while others contained more cyclic features. The binding energy range for the top 10 was −12.2 kcal/mol to −14.8 kcal/mol, which is a stronger binder than that of the endogenous ligand with binding energy of −11.1 kcal/mol. The majority of the PFAS had nonpolar features, showing minimal other elements other than carbons and fluorines, but were taken apart on many different conformations, like bicyclic features. The results feature the potential risks PFAS may pose to the function of ERα. At the orthosteric site of ERα, 40 PFAS demonstrated greater binding affinity than estradiol, demonstrating that some PFAS may have greater binding affinity than endogenous ERα ligands.

#### 2.2.8. PFAS Virtual Screening: ERα Allosteric Site

The range for the top 10 PFAS bound at the ERα allosteric site was −9.8 kcal/mol to -14.1 kcal/mol (Table S10). A majority of the top PFAS display more structural similarities than those at the orthosteric site, as most contain cyclic features. The top 10 ligands do not have many differing functional groups and just have carbon and fluorine bonds for the most part. The allosteric site appears to be more nonpolar than the orthosteric site, as there are more variable functional groups in the PFAS that bind to the orthosteric site rather than those that bind to the allosteric site. A total of 7 PFAS displayed a greater docking score than SRC-1 at the allosteric site, demonstrating the strength in which PFAS bind to the allosteric site of ERα.

#### 2.2.9. Competitive Binding Analysis of PFAS With Nuclear Receptor Native Ligands

Next, we sought to assess the average binding strengths and the potential of PFAS chemical competitive interaction with native ligands of nuclear receptors (NRs). We compared the docking scores of PFAS at both orthosteric and allosteric sites of various NRs.

- Competitive Interaction Analysis at the Orthosteric Site.

**Figure 11:**
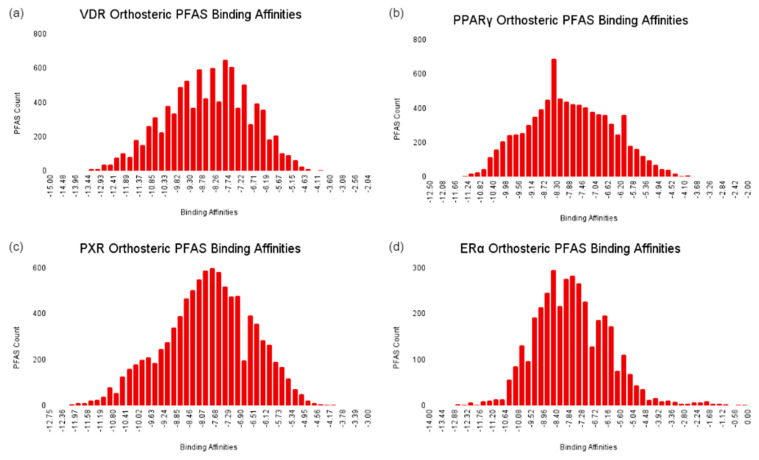
The distributions of PFAS binding affinities measured in kcal/mol are displayed at the orthosteric site of (**a**) PXR, (**b**) PPARγ, (**c**) ERα, and (**d**) VDR.

The distribution of PFAS docking scores at the orthosteric sites exhibited a normal distribution for all NRs, as displayed in Figure 11. For the VDR, the mean docking score for PFAS was −8.6 kcal/mol, which is weaker compared to the −12.3 kcal/mol docking score of the native ligand, calcitriol. This suggests that most PFAS possess a moderately lower risk of competitive binding at the VDR. However, it should be noted that 130 PFAS displayed a docking score higher than calcitriol, highlighting a strong risk of competitive binding for these specific PFAS compounds. At the PPARγ, PFAS achieved a mean docking score of −7.9 kcal/mol, in contrast to −9.0 kcal/mol for the native ligand Indeglitazar. The 1.1 kcal/mol difference in binding affinity emphasizes a strong risk of competitive binding. The strong risk of competitive binding is further emphasized by the 1823 PFAS that displayed docking scores greater than Indeglitazar. For the PXR, the mean PFAS docking score was −7.9 kcal/mol, compared to −10.2 kcal/mol for the reference ligand, indicating a moderate risk of competitive binding for most PFAS. Nonetheless, 650 PFAS displayed a docking score greater than the reference ligand, underscoring a considerable risk of competitive binding at the PXR site for these compounds. At the ERα, the mean docking score of PFAS was −8.6 kcal/mol, lower than the −11.1 kcal/mol score for estradiol, suggesting a moderate potential for competitive binding. Despite this, 40 PFAS displayed docking scores greater than estradiol, emphasizing a notable risk of competitive binding for these specific PFAS compounds at the ERα site.

- Competitive Interaction Analysis at the Allosteric Site.

**Figure 12:**
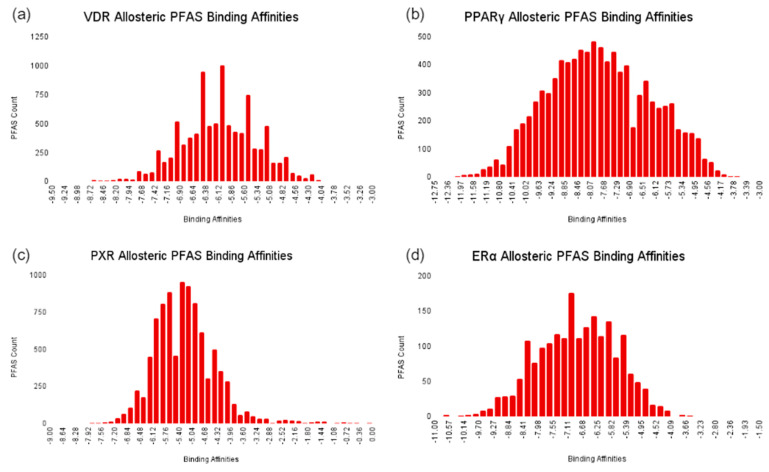
The distributions of PFAS binding affinities measured in kcal/mol are displayed at the allosteric site of (**a**) PXR, (**b**) PPARγ, (**c**) ERα, and (**d**) VDR.

Similar to the orthosteric sites, docking scores at the allosteric sites of NRs showed a normal distribution, as depicted in Figure 12. At the VDR allosteric site, PFAS demonstrated a mean docking score of −6.1 kcal/mol, which is slightly lower than −6.6 kcal/mol for the native ligand lithocholic acid (LCA), indicating a significant potential for competitive binding. This potential for competitive binding is further evidenced by 2229 PFAS displaying higher affinities than LCA. The mean docking score of PFAS at the allosteric site of PPARγ was −7.8 kcal/mol, which is lower than the −10.0 kcal/mol observed for T35, suggesting a decreased potential of competitive binding compared to the orthosteric site. However, 693 PFAS bind stronger than T35, highlighting that there is still a notable risk of competitive binding at the PPARγ allosteric site. At the PXR allosteric site, PFAS had a mean score of −6.0 kcal/mol, marginally higher than the −5.6 kcal/mol score for glycerol, which highlights a significant risk of competitive binding at this site. Remarkably, 9148 PFAS displayed higher affinities than glycerol, further emphasizing this risk. For ERα, PFAS showed a mean docking score of −6.1 kcal/mol, considerably lower than the −9.9 kcal/mol for SRC-1, indicating a low potential for competitive binding at this site.

Taken together, this analysis reveals varying levels of competitive binding potential for PFAS across different NRs and binding sites, which provide some insights for understanding the implications of PFAS exposure on the regulation of endocrine signaling pathways and adverse health effects such as cancer.

### 2.3. Commercially Relevant PFAS Molecule Virtual Screening: VDR

Virtual screening was performed on the commercially relevant and commonly exposed PFAS for the VDR as a test case study to understand their binding potential and impacts. PFOA and PFOS are exposed to humans at higher concentrations compared to other PFAS [32]. PFOA and PFOS have been linked with the suppression of nuclear receptors like HNF4α [33]. Childhood diseases and diminished antibody response have been correlated with greater PFOA and PFOS exposure [34]. PFOA and PFOS have been associated with cancer through mechanisms such as DNA/RNA damage, lipid peroxidation, and cell death [35]. More specifically, PFOS has been shown to interact with a lung surfactant in effects to result in lung collapse and failure, whereas PFOA has been associated with human cell apoptosis, and increasing studies are relating it to human carcinogenicity [36,37]. The further analysis shows that PFOS and PFOA can display a similar or higher binding affinity to the VDR orthosteric site, regardless of its lower-than-calcitriol individual binding affinity. In the allosteric site, high concentration and binding strength play a role for VDR overactivation or dysregulation caused by PFOA and PFOS. Other than PFOA and PFOS, other commercially relevant PFAS include Perfluorononanoic acid (PFNA), Tridecafluorohexane-1-sulfonic acid (PFHxS), Perfluoroheptanoic acid (PFHpA), and Perfluorodecanoic acid (PFDA). PFNA, PFDA, PFHpA, and PFHxS are similar to PFOA and PFOS, and have similar effects to the orthosteric and allosteric sites of the NRs. Previous studies on PFDA reported the associated effects with increasing gastric cell proliferation and the suppression of cell senescence [38]. In higher concentrations, PFNA has shown to increase rates of melanoma in women and chances of uterine cancer. Germ cell tumor responses have a relationship with increased PFHxS concentration [39].

#### 2.3.1. Commercially Relevant PFAS Virtual Screening: VDR Orthosteric Site

The binding strength of the commercially relevant PFOA and PFOS was analyzed. At the orthosteric site, PFOA is shown to have a docking score of −8.6 kcal/mol while PFOS is −9.6 kcal/mol. By docking score, PFOA and PFOS are not competitive to the orthosteric site of the VDR as calcitriol has a docking score of −12.3 kcal/mol. Previous research indicates that PFOA and PFOS exposure does not lead to dysregulation to the fullest extent on the VDR. However, that is not limited to other PFAS, as other PFAS types show greater binding affinity to the VDR than PFOS and PFOA [14]. Since PFOA and PFOS do not display a greater affinity for the VDR in comparison to calcitriol, the contributing factor for modified receptor function is due to its high concentration exposure. Alongside PFOA and PFOS, other common PFAS that showed significance to humans are PFDA, PFNA, PFHxS, and PFHpA. PFDA had the greatest binding affinity as the docking score was −9.7 kcal/mol, the strongest binding among the relevant PFAS (Table S11). Similarly strong was PFNA, which was predicted to have a relatively strong docking score of −9.6 kcal/mol, which is the same as PFOS. PFHxS and PFHpA had the lowest docking scores with −8.3 kcal/mol and −8.2 kcal/mol, respectively. With lower docking scores, commercially relevant PFAS have been shown to affect receptor functionality due to their high concentrations. PFDA has the greatest binding affinity along with the higher exposure possibility to humans that increases its chance to bind to the VDR. However, it is important to note that there are 130 different PFAS chemical classes that can bind to the VDR’s orthosteric site, and they can also be found in commercial products, though not as prevalent as the ones listed above. While the commercially significant PFAS have lower docking scores than calcitriol at −12.3 kcal/mol, the prevalence and higher exposure concentration of these relevant PFAS can induce carcinogenic effects through VDR.

#### 2.3.2. Commercially Relevant PFAS Virtual Screening: VDR Allosteric Site

At the allosteric site, PFOS and PFOA both displayed a docking score of −6.7 kcal/mol, greater than the docking score of lithocholic acid at −6.6 kcal/mol. Lithocholic acid plays a protective role for TNF-α-induced injury of the intestinal barrier by binding to the VDR [40]. LCA has proven to bind at both the LBD and allosteric site, but when binding to the allosteric site, it provides more structural balance for the VDR. The previous study, targeting the allosteric site, showed that LCA binds to helix 12, therefore stabilizing the active agonist conformation [19]. Given the elevated concentrations of PFOA and PFOS, it can bind to the VDR allosteric site as well, altering the activity of the receptor. PFDA, PFNA, PFHxS, and PFHpA were also bound in the allosteric site, and showed more significant binding affinity than in the orthosteric site (Table S11). PFDA and PFNA had the greatest binding affinity among these four relevant PFAS at -6.8 kcal/mol and −6.7 kcal/mol, respectively. They both displayed greater binding affinity than lithocholic acid, which had a docking score of −6.6 kcal/mol. This is significant as the activity of the VDR can be altered as LCA plays a role in regulating the VDR functions. PFDA and PFNA in high exposure can cause VDR activation or dysregulation once bound to the allosteric site, due to its greater binding affinity and concentration. PFHxS and PFHpA have docking scores of −6.4 kcal/mol and −6.5 kcal/mol, which is just under the docking score of LCA. Due to PFHxS and PFHpA having binding affinities that are only lower than LCA by 0.1–0.2 kcal/mol, their individual binding affinity is not significantly weaker than LCA. This means that they can be competitive binders to the allosteric site. In combination with the high concentration of PFHxS and PFHpA, the allosteric site of the VDR is very susceptible to being regulated by commercially relevant PFAS.

### 2.4. Modeling of PFAS Mixture Binding to the Nuclear Receptor

Exogenous chemicals, including hydrophobic compounds, have shown great potential in interacting with the binding pockets of NRs. These interactions have been shown to cause activation and dysregulation, resulting in carcinogenic outcomes. Additionally, NR overactivation can also occur when hydrophobic exogenous chemicals engage with the receptor [19]. Distinct combinations of these non-endogenous chemicals binding to nuclear receptors have been identified. We reviewed seven significant combinations for mixture modeling between endogenous and exogenous chemicals. Due to the uniqueness of each PFAS class, the possible case scenarios for mixture modeling including PFAS are significant. The first case scenario occurs when the endogenous ligand occupies the orthosteric site concurrently with an exogenous chemical to the allosteric site. Another scenario exists when an endogenous ligand binds at the allosteric site simultaneously with an exogenous chemical at the orthosteric site. Lastly, a scenario arises when both the orthosteric and allosteric sites of the receptor are engaged by non-native ligands. This scenario supports that NR overactivation can occur when two exogenous ligands simultaneously bind to the receptor, as the hydrophobicity brings stabilization, and the effect causes a super-activated receptor [19]. Under this category of activation, there are subcategories as different PFAS classes alter their induced effects, which are discussed below.

**Figure 13:**
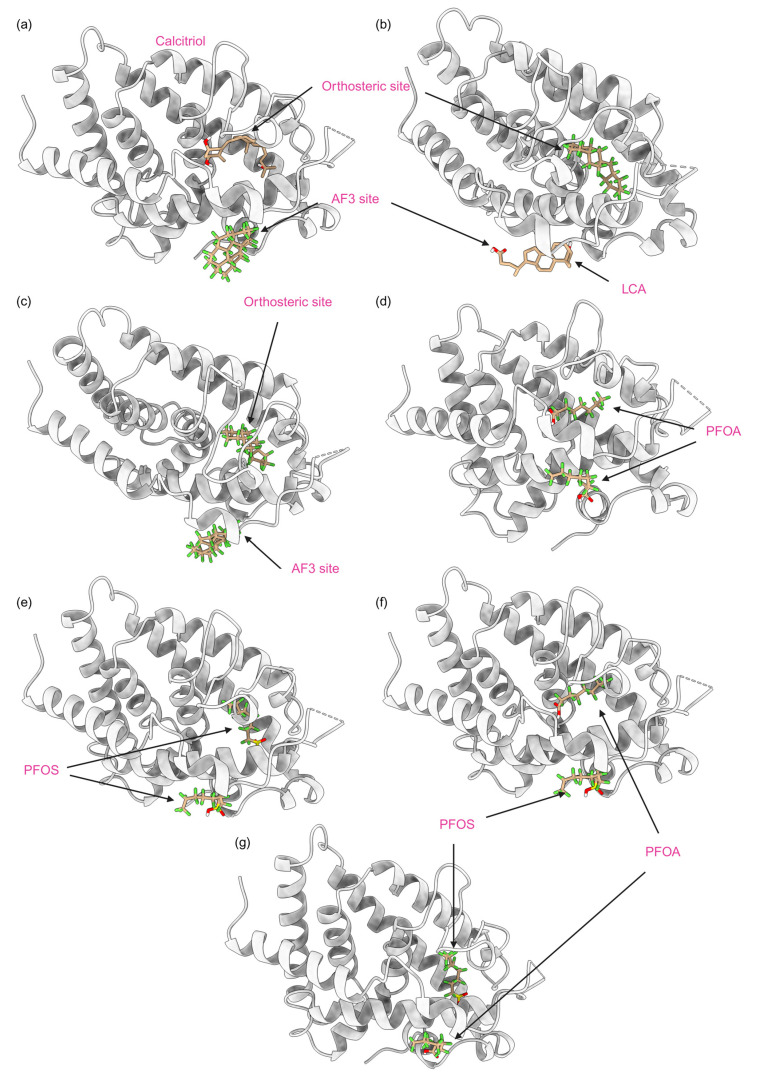
(**a**) The top PFAS by docking score at the allosteric site is displayed, Hexatriacontafluorotetracosahydrocoronene, under the classification of Polycyclic Perfluoroalkane. (**b**) The top PFAS by docking score at the orthosteric site is displayed, Perfluoroperhydrobenzyl tetralin, under the classification of Polycyclic Perfluoroalkane. (**c**) The top PFAS at both the orthosteric and allosteric sites are displayed, Perfluoroperhydrobenzyl tetralin and Hexatriacontafluorotetracosahydrocoronene, respectively, and both are under the classification of Polycyclic Perfluoroalkane. (**d**) Perfluorooctanoic acid is displayed at the orthosteric and allosteric site of the VDR, and the PFAS class is perfluorinated alkyl acids, and more specifically carboxylic acids. (**e**) Perfluorooctane sulfonic acid is displayed at the orthosteric and allosteric site of the VDR, and the PFAS class is perfluorinated alkyl acids, and more specifically sulfonic acids. (**f**) Perfluorooctanoic acid is displayed at the orthosteric and perfluorooctane sulfonic acid is displayed at the allosteric site of the VDR. (**g**) Perfluorooctane sulfonic acid is displayed at the orthosteric and perfluorooctanoic acid is displayed at the allosteric site of the VDR.

In this study, we report only the results of the simultaneous binding of non-native ligands, i.e., PFAS and native ligands binding to the VDR. Figure 13 visualizes various mixed models with seven significant combinations of PFAS and endogenous or native ligands, each influencing the activation or altered function of the VDR. PFAS may have toxic effects when bound simultaneously to the allosteric site in combination with the calcitriol at the orthosteric site, potentially causing an overactivation of the VDR as shown in Figure 13a). In this model, lithocholic acid (LCA) can be substituted by the PFAS DTXSID70597457 (Fluoropolymer—FP) at the allosteric site, while the orthosteric site remains occupied by calcitriol. This mode of receptor activation requires PFAS binding to the allosteric site, which, due to its lower binding affinity and more superficial location compared to the orthosteric site, is more accessible, allowing various PFAS to bind and potentially cause receptor overactivation. The combination of PFAS at the allosteric site with calcitriol at the orthosteric site may pose a potential hazard, as the PFAS stabilizes the VDR while calcitriol performs its native function, potentially resulting in an overactivated VDR complex. For example, as shown in Figure 13, panel b, LCA binds to the allosteric site while a high-energy binding PFAS, DTXSID40881335 (Fluoropolymer—FP), occupies the orthosteric site. These combinations are common due to the hydrophobic nature of PFAS and the abundance of endogenous ligands such as calcitriol and LCA. Consequently, one endogenous ligand will bind to the receptor while the other site is occupied by a PFAS, leading to the overactivation of the VDR. In scenarios of high PFAS exposure, two PFAS molecules could bind to both the orthosteric and allosteric sites, as illustrated in Figure 13, panel c, with two high-affinity binding PFAS, including DTXSID40881335 at the orthosteric site and DTXSID70597457 at the allosteric site. Both PFAS molecules, consisting only of carbon–fluorine rings and chains, enhance the hydrophobic interaction, which is highly carcinogenic due to the non-endogenous nature of these chemicals, resulting in significant protein stabilization and super-activation. Particularly concerning are PFAS commonly found in humans, such as perfluorooctanoic acid (PFOA) and perfluorooctane sulfonate (PFOS), which are often found at high concentrations. As depicted in Figure 13, panels d through g, PFAS chemicals can occupy both the orthosteric and allosteric sites of the VDR, leading to overactivation that could lead to carcinogenesis. This is significant given the prevalent rates of PFOA and PFOS in humans, which could lead to VDR overactivation and potential cancer risks.

These super-activation mechanistic scenarios are similarly applicable to other nuclear receptors such as ERα, PXR, and PPARγ. Previous investigations demonstrated the super-activation of the VDR by the motor fuel oil compound Salpn, which binds to the allosteric site, enhancing stability and inducing excessive agonistic activity when combined with calcitriol [19]. Similarly, PFAS molecules, due to their hydrophobic nature similar to endogenous hormone ligands, exhibit strong affinities for both allosteric and orthosteric sitesof nuclear receptors. This interaction has been implicated in the overproduction of hormones, particularly in the case of ERα, potentially leading to carcinogenic effects similar to those observed with exposure to carcinogens [26]. PFAS have shown effects like altering receptor function when bound with calcitriol to the VDR, creating a super-agonistic effect [5,19]. The ligand binding domain (LBD) orthosteric site has a large volume that can accommodate both calcitriol and PFAS, presenting another possibility of overactivation where both ligands occupy the orthosteric site; however, this scenario is not explored in the context of this study. Nevertheless, the structural similarities between the orthosteric and allosteric sites of nuclear receptors allow for the prediction that PFAS could have similar overactivation effects on other nuclear receptors as observed in the VDR by Salpn [5,19].

### 2.5. Comparison to Previous Molecular Docking Studies

Molecular docking studies have previously studied the binding interactions between PFAS and nuclear receptors. The docking software used in this study, Vina 1.2.5, has been used to investigate perfluorinated alkyl acids with three protein complexes [41] (Ng et al., 2015). Other studies have used different molecular docking software such as DiffDock to predict PFAS–protein interactions [42]. This study focuses on PFAS binding at two sites of nuclear receptors, specifically VDR, PXR, PPARγ, and ERα, to understand their possible mechanism in inducing carcinogenic responses. The novelty of this study is attributed to the large scale of PFAS that have been screened, over 9000, and analyzing the binding interactions at the orthosteric and allosteric sites of the specific NRs. Additionally, the uniqueness of this study stands in the exploration of simultaneous binding of native and non-native PFAS ligands at the orthosteric and allosteric sites. Further, the understanding of the possible mechanisms where PFAS can induce carcinogenesis can be revealed via in vitro or in vivo experimentation. However, the goal of this study is to obtain a potential array of PFAS that can bind to either or both binding sites of NRs in this study. PPARγ and ERα have been previously studied and docking simulations have been conducted with over 6000 PFAS, over 3000 less than our study [43]. Similarly, the potential binding of 5206 PFAS to VDR has been investigated using molecular docking [14]. Another in silico-based screening investigated PPARγ with 43 PFAS in order to investigate how the structure of PFAS impacts binding potency [44]. Computational techniques, such as molecular dynamics, were previously used to assess the binding strength between PFOS, PFOA, and PPARγ [45]. This study revealed strong binding between PFOA and PPARγ. Given the significant differences in the number of PFAS investigated, it would be difficult to compare results and obtain any meaningful insights. Furthermore, the allosteric binding sites of the NRs have been seldom studied with PFAS, thus proving difficult to compare results.

## 3. Methods and Materials

### 3.1. Overall Methodology Workflow

To begin the docking process, the structures of VDR, PXR, PPARγ, and ERα were obtained and prepared. The structure of PFAS was downloaded from the EPA CompTox database and subsequently ligands were prepared for docking. To confirm the docking protocol, endogenous ligands were docked and their binding conformations were verified and confirmed with many native and nonligand docking as controls. Molecular docking for the PFAS compounds was then performed. The binding affinities were then ranked and the PFAS with stronger binders were analyzed and investigated. A more detailed methodology workflow can be found in Figure S4.

### 3.2. Receptor and Native Ligand Preparation and Docking Simulations

The orthosteric and allosteric structures were retrieved from the Protein Data Bank for VDR, PXR, PPARγ, and ERα (Table S12). Using ChimeraX 1.7.1 [46,47], all water molecules and ligands were removed from the receptor. Subsequently, these receptor files were processed with Autodock 4/4.2 Tools [48], adding polar hydrogens to facilitate intermolecular bonding with ligands, merging of nonpolar hydrogens, the assignment of Kollman charges, and setting the pH to 7. The Kollman charges were selected; they are calculated using the AMBER force field and are based on a molecule’s molecular orbital electron density, which makes them more suitable for accurately representing proteins and other large biological molecules. Subsequently, the protein was exported in .PDBQT format, for Autodock Vina.

All ligands were reprocessed using the software Gypsum-DL 1.2.1 [49]. Smiles (.smi) versions of the ligands were inputted, and 3D .sdf output files of the ligands were obtained. A python code was created to convert the 3D .sdf files into 3D .PDBQT files that was optimized for the best conformer structure (Supplementary File Python Code). In preparation for docking, Autodock tools were used to locate the allosteric and orthosteric sites. The grid box for each receptor was created (Table S13).

The docking was restricted within the orthosteric and allosteric sites of the VDR, PXR, PPARγ, and ERα to obtain a greater success rate [50]. To identify the accuracy of the docking, and for the controlled simulations, endogenous ligands were redocked in their respective binding domains. For the VDR receptor, calcitriol and lithocholic acid molecules were redocked onto the orthosteric and allosteric site, respectively, using Autodock Vina (Vina) [51]. Similarly, redocking of endogenous ligands for PPARγ, PXR, and ERα was carried out.

### 3.3. PFAS Preparation and Docking

The chemical structures for the PFAS were downloaded from the Environmental Protection Agency (EPA) CompTox chemical dashboard in June 2023 (https://www.epa.gov/comptox-tools/comptox-chemicals-dashboard accessed on 6 June 2023). Starting with 12,034 chemicals, the PFAS that had no structures, multiple structures, and isotopes were filtered out. The DTX SID was used as a way of differentiating the PFAS chemicals, as it is the chemical identifying method for CompTox. The chemical structures of PFAS were downloaded as SDF files. Using OpenBabel 2.3.3, the SDF file was converted to a SMILES file containing all the PFAS chemicals with SMILES codes. The PFAS chemicals without proper SMILES codes were discarded and removed from the dataset. A total of 9870 PFAS chemicals were prepared using Gypsum-DL by converting the 2D PFAS structures into accurate 3D compounds. In addition to generating the 3D structures, the compounds were neutralized at a pH of 7. The 9870 compounds successfully prepared using Gypsum-DL were energy-minimized using obminimize from OpenBabel 2.3.3 and, using the general amber force field (GAFF) with default parameters, and were then converted into PDBQT files. The Python code (Supplementary File Python Code) was capable of optimizing the conformations of all ligands using the AMBER force field and the Obminimize capability from OpenBabel 2.3.3 The optimization process, which resulted in the generation of PDBQT output files for each PFAS, allowed for the use of molecular docking with the Vina 1.2.5 software.

### 3.4. Molecular Docking

Molecular docking simulations were conducted using Vina, employing the AMBER force field. The simulations involved interactions between proteins, native ligands, and PFAS. Specifically, the docking simulations were focused on the orthosteric and the allosteric sites with the corresponding endogenous ligand. Grid box coordinates were created as described above in Section 2.2. A Vina-based virtual screening was executed for 9870 PFAS molecules onto the orthosteric site. Following the completion of the orthosteric site, the grid box parameters were adjusted to target the allosteric site where the endogenous ligand binds, and all PFAS molecules were subsequently docked onto the allosteric site. The docking protocol and the accuracy of Vina predictions were verified using cross-docking of native ligands at both the orthosteric and allosteric sites for the VDR, PXR, ERα, and PPARγ. The docking of native ligands at both orthosteric and allosteric sites of the NRs resulted in accurate binding conformations within 2 Å of their reference pose, highlighting the accuracy of Vina in predicting the protein–ligand interactions.

## 4. Strengths and Limitations

This PFAS virtual screening study is distinct from others as it investigates over 9000 PFAS chemical interactions with PPARγ, PXR, VDR, and ERα, at their orthosteric and allosteric sites. The interactions between PFAS and the allosteric sites of these nuclear receptors (NRs) are particularly a strength, as they include more possibilities for receptor activation. Further, the inclusion of allosteric sites is significant, as the results obtained in this study demonstrate that PFAS can readily bind these sites due to easier access and lower binding potential. In NRs, the allosteric site plays a major role in maintaining receptor stability, contributing to homeostatic responses. However, when occupied by exogenous ligands, hyperactivation can occur, underscoring the importance of studying the allosteric site [5,19,52]. Mixture models of PFAS were developed in combination with other PFAS and endogenous ligands to illustrate how NRs can be overactivated. Binding of PFAS to either the orthosteric or allosteric sites of nuclear receptors can result in hyperactivation, similar to the effects observed with carcinogenic compounds. Different classes of PFAS may also alter the activation status of the nuclear receptor. For example, in the context of the VDR, simultaneous binding of PFAS and endogenous ligands can lead to receptor stability and hyperactivation, reflecting carcinogenic pathways. In the earlier Salpn–VDR study, Salpn has been associated with the overactivation of other nuclear receptors, including PPARγ, androgen receptor (AR), ERα, and thyroid hormone receptor alpha (TRα) [19]. The hydrophobicity of Salpn, similar to that of PFAS, supports the conclusion that PFAS can have analogous effects on nuclear receptors when bound. It is noteworthy that some of these mixed model scenarios are hypothetical and require further validation. However, these mixture models provide valuable representations of how the combinatorial effects of PFAS can lead to different types of functional alterations in the receptor. In this study, the local binding site flexibility was considered; however, future investigations are needed to include full flexibility of the protein and ligand complex. Fully flexible docking will offer more precise predictions and provide additional insights into how PFAS can bind with NR complexes. Further improvements to this study would involve employing MM-GBSA or MM-PBSA methods to assess the full-fledged binding free energy of top PFAS ligands to the NRs. This approach would enhance the accuracy of the computational model. Additionally, expanding the scope of this study beyond PFAS to understand how other chemicals, both standalone and in combination with PFAS, can induce carcinogenic responses is important. This broader examination is important for understanding the full range of toxicological impacts and potential health risks posed by chemical interactions with nuclear receptors. In vitro experiments should be performed to validate the results obtained in this study. The strong binders identified for VDR, PXR, PPARγ, and ERα should be investigated to determine if they can activate their respective nuclear receptors. We are progressing toward obtaining these results as future directions.

## 5. Future Directions

Ongoing studies are underway to understand how PFAS and other endocrine disrupting chemicals (EDCs) in combination can cause the overactivation of NRs. This can be performed by the mixture modeling, as conducted in this study for the VDR and earlier mixed model study [19], for EDCs and PFAS, which will display how they can cause overactivation simultaneously. EDCs such as polybrominated diphenyl ethers (PBDEs), microplastics (MPs), nanoplastics (NPs), and pesticides including pesticides and nonpersistent EDCs like parabens, phenols, and phthalates have shown carcinogenic effects [53]. Analyzing the combinatorial effect of PFAS and EDCs is relevant due to the carcinogenic nature of both chemicals. Similar to PFAS, EDCs and nonpersistent EDCs have been correlated with exposure to humans through consumption, and causing nuclear receptor dysregulation [54]. Studying PFAS alongside pesticides and EDCs holds importance for future research, given their common presence in humans, according to the latest report by the Pesticide Action Network (PAN). Their potential to induce cancerous effects emphasizes the significance of the analysis of their combinatorial effect. Studies have shown EDCs’ effects on nuclear receptors such as PPARγ, ERα, and PXR, to have detrimental effects at low concentrations [55], raising the concern when combined with PFAS, with disrupting capabilities to nuclear receptors, resulting in super-activation and carcinogenesis. Therefore, analyzing the effects PFAS, pesticides, and EDCs induce together is important for understanding how they can cause carcinogenic effects.

## 6. Conclusions

Large-scale screening of over 9000 PFAS was performed using computational docking simulations. The binding affinities were analyzed and the binding strength of PFAS with nuclear receptors such as VDR, PXR, PPARγ, and ERα was evaluated. The binding affinities were quantified in kcal/mol, and PFAS were subsequently ranked based on these values. Numerous PFAS exhibited binding energies higher than corresponding endogenous or native ligands. These strong interactions between PFAS and the nuclear receptors were influenced by inherent hydrophobic interactions between PFAS and the NRs. Despite diverse interactions of PFAS across different receptors due to varied amino acid residues within the binding domains, commonalities persist in the hydrophobic nature of these interactions. Notably, the top binding PFAS at orthosteric sites of PXR, VDR, and ERα were predominantly hydrophobic, consisting mainly of carbon and fluorine, whereas top binders to PPARγ included additional elements, reflecting less hydrophobicity in the ligand binding domain (LBD) of PPARγ compared to other receptors. Unlike other NRs studied here, PPARγ had a smaller count of stronger binding PFAS in comparison to the endogenous ligand at the allosteric site relative to the orthosteric site. The results obtained on the allosteric site of these NRs highlight the novelty of this study, as the interactions between these alternative binding sites and PFAS have been understudied. Through large-scale screening, a significant number of PFAS were identified that appear to have strong binding interactions with these allosteric sites, which could potentially elucidate the mechanisms in which PFAS dysregulated VDR, PXR, PPARγ, and ERα.

Further investigation on the binding affinity of commercially relevant PFOA and PFOS, towards the VDR, demonstrates that PFOA and PFOS do not effectively displace or compete with calcitriol, at the orthosteric site, a primary binding site on the VDR. In contrast, PFAS can compete with the allosteric ligand lithocholic acid (LCA), a bile acid, and can easily displace it from its binding position due to its relatively lower affinity. Additionally, PFAS structurally mimic the bile acid and exhibit highly similar physicochemical properties, forming stronger binding interactions with the VDR at the allosteric site. This displacement allows the stable PFAS to bind more readily to secondary, allosteric sites on the VDR. Such allosteric binding does not hinder the primary activation of the receptor by calcitriol but contributes to the additional affinity and influence receptor conformation, leading to the activation. Moreover, the higher concentrations of these PFAS significantly enhance their likelihood of interacting with the VDR. This concentration-dependent binding suggests that environments with elevated PFAS levels, such as in industrial or contaminated sites, could be at an increased risk of these substances interacting with the VDR or other nuclear receptors, potentially leading to altered receptor activity and downstream biological effects.

Several mixture combinations of PFAS, calcitriol, and LCA against the nuclear receptors were developed to analyze simultaneous binding potential at their orthosteric and allosteric sites. In the case of VDR, the mixed models suggest that some PFAS can hyperactivate the VDR by the synergistic effects through simultaneous binding at their orthosteric and allosteric sites. The continued studies into the combinatorial effects of PFAS are ongoing. This is important, specifically, given the persistent and rising environmental concentrations of these substances and their hazardous effects to humans.

While this study provides significant insight into the interactions of PFAS with VDR, PXR, PPARγ, and ERα, further research, particularly in vitro research, should be conducted to better understand these interactions. Therefore, we call for PFAS that have been identified as strong binders to be more conclusively studied to further understand the carcinogenic effects that PFAS binding to NRs can cause. We cannot ignore the unwarranted exposure of PFAS in foods, water, cosmetics, etc. Thus, we advocate for policy changes that incorporate more research into these forever chemicals.

## Figures and Tables

**Table 1:** Docking scores for crossed docked ligands in the orthosteric and allosteric sites of VDR are shown, and all appear at a lower docking score than calcitriol and lithocholic acid, which has a docking score of −12.3 and −6.6, respectively.

Protein	Ligand	Orthosteric Docking Score	Allosteric Docking Score
Farnesoid X Receptor	Chenodeoxycholic	−9.4	−6.4
Estrogen Receptor	Estradiol	−9.4	−6.5
Retinoic Acid	Tretinoin	−9.1	−5.9
PPARG	15-deoxy-Δ-12,14-Prostaglandin	−8.2	−4.9
Transthyretin	Triiodothyronine	−8	−5.3

**Table 2:** Classification of Top PFAS Compounds for PPARγ Orthosteric Site.

DTXSID	CASRN	Preferred Name	Classification
DTXSID20816403	61547-75-9	N,N′-Bis[2-(1,1,2,2-tetrafluoro-2-phenylethyl)phenyl]urea	Perfluorocarboxamide
DTXSID201033555	749924-57-0	1-(9H-Fluoren-2-yl)-perfluoro-1-butanone O-[(perfluorobutyl)sulfonyl]oxime	Perfluoroalkane sulfonamido substances
DTXSID60371061	231953-37-0	1-Benzoyl-3,5-bis(heptafluoropropyl)pyrazole	Perfluoro carboxamide
DTXSID401026885	NOCAS_1026885	4-Fluoro-N-[1-(4-fluorophenyl)-1-hydroxy-3-(2,2,3,3-tetrafluoro-1,4-benzodioxin-6-yl)propan-2-yl]naphthalene-1-carboxamide	Perfluoroethers
DTXSID90776848	1555-24-4	2,5-Bis(pentadecafluoroheptyl)-1,3,4-oxadiazole	Perfluorooxadiazole
DTXSID50238598	915-76-4	2,4,6-Tris(heptafluoropropyl)-1,3,5-triazine	Perfluorotriazine
DTXSID80198893	5086-79-3	1,3-Bis(2-phenyl-1,3,4-oxadiazol-5-yl)perfluoropropane	Perfluorooxadiazole
DTXSID50290777	4368-75-6	3,3′-(1,1,2,2,3,3-hexafluoropropane-1,3-diyl)bis[5-(pentafluoroethyl)-1,2,4-oxadiazole]	Perfluorooxadiazole
DTXSID601035822	NOCAS_1035822	Perfluoro-13-(pentafluoro-lambda6-sulfanyl)-1-tridecanesulfonic acid	Perfluoroalkane sulfonic acid
DTXSID30896453	862130-87-8	2-Chloro-4-fluoro-N-(3-{[4-(1,1,1,2,3,3,3-heptafluoropropan-2-yl)-2,6-dimethylphenyl]carbamoyl}phenyl)benzamide	Perfluorocarboxamide
DTXSID60896168	151707-04-9	1,1′-[1,3-Phenylenebis(oxy)]bis[3-(tridecafluorohexyl)benzene]	Perfluoroether
DTXSID30897041	57912-15-9	(Perfluoropropyl)-2-(2-phenylhydrazinylidene)ethanal phenylhydrazone	Perfluoro something nitrogen
DTXSID90896459	862130-95-8	2,3,6-Trifluoro-N-(3-{[4-(1,1,1,2,3,3,3-heptafluoropropan-2-yl)-2,6-dimethylphenyl]carbamoyl}phenyl)benzamide	Perfluorocarboxamide
DTXSID601020695	786629-35-4	4-((Henicosafluorodecyl)oxy)benzenesulfonic acid	Perfluoroether and perfluoro sulfonic acid
DTXSID00768057	132877-69-1	1,4-Bis(heptadecafluorooctyl)benzene	Perfluoroalkanes

**Table 3:** Docking scores for top 15 significant PFAS that have a greater docking score than calcitriol at the VDR orthosteric site.

DTXSID	Preferred Name	CASRN	Docking Score
DTXSID40881335	Perfluoroperhydrobenzyl tetralin	116265-66-8	−14.7
DTXSID60881337	Perfluoro(4a-(cyclohexylmethyl)decahydronaphthalene)	125061-94-1	−14.5
DTXSID101023399	Cyclohexane, 1,1,2,2,3,3,4,4,5,5,6-undecafluoro-6-(1,1,2,2,3,3,4,4,5,5,6,6,7,7,8,8,9,9,10,10,10-heneicosafluorodecyl)-	87667-00-3	−13.6
DTXSID701036930	2-(Perfluoroeicosanyl)ethane-1-sulfonic acid	–	−13.4
DTXSID60895499	Hexatetracontafluorodocosane	1127427-64-8	−13.4
DTXSID901009913	Perfluorooctadecanesulfonic acid	51604-60-5	−13.4
DTXSID40798070	2,2′-Dimethoxy-5,5′-bis(tridecafluorohexyl)-1,1′-biphenyl	658709-63-8	−13.4
DTXSID301033158	Perfluorohenicosanoic acid	–	−13.3
DTXSID901026642	Tritriacontafluoroheptadecanoate	–	−13.2
DTXSID00768057	1,4-Bis(heptadecafluorooctyl)benzene	132877-69-1	−13.2
DTXSID00379561	Methyl perfluorooctadecanoate	16753-33-6	−13.2
DTXSID301034649	1-Hydroxy-18:2 fluorotelomer sulfonic acid	–	−13.2
DTXSID1047029	Perfluorotetradecahydrophenanthrene	306-91-2	−13.2
DTXSID10880471	(1H,1H-Perfluoro-16-methylheptadecyl)oxirane	54009-79-9	−13.2
DTXSID7070218	(Perfluorooctadecyl)ethyl 2-propenoate	65104-64-5	−13.2

**Table 4:** Docking scores for top 15 significant PFAS that have a greater docking score than lithocholic acid at the VDR allosteric site.

DTXSID	Preferred Name	CASRN	Docking Score
DTXSID70597457	Hexatriacontafluorotetracosahydrocoronene	51344-02-06	−10.4
DTXSID60881337	Perfluoro(4a-(cyclohexylmethyl)decahydronaphthalene)	125061-94-1	−9.9
DTXSID40881335	Perfluoroperhydrobenzyl tetralin	116265-66-8	−9.8
DTXSID90984683	Hexacosafluorohexadecahydrofluoranthene	662-28-2	−9.8
DTXSID501041016	Cyclohexanecarboxamide, N,N′-[4-(phenylazo)-1,3-phenylene]bis[1,2,2,3,3,4,4,5,5,6,6-undecafluoro- (9CI)	548470-06-0	−9.6
DTXSID1047029	Perfluorotetradecahydrophenanthrene	306-91-2	−9.6
DTXSID90380005	Perfluoroperhydrofluorene	0307-08-04	−9.4
DTXSID601006972	Perfluoro-N-(4-methylcyclohexyl)piperidine	86630-50-4	−9
DTXSID70880119	Perfluoro-1,2-bis(perfluorocyclohexyl)ethane	306-99-0	−9
DTXSID30952942	Icosafluorododecahydroacenaphthylene	0307-07-03	−8.9
DTXSID40880121	Perfluoro-2-methyldecalin	306-95-6	−8.8
DTXSID10449906	Peroxide, bis[(undecafluorocyclohexyl)carbonyl]	203255-90-7	−8.8
DTXSID30897561	1-(Trifluoromethyl)perfluorodecalin	306-92-3	−8.7
DTXSID80893316	Perfluoro-5,5′-bis(trifluoromethyl)-1,1′-bicyclohexyl	105462-77-9	−8.7
DTXSID10539058	1,1,2,2,3,3,4,4,6,6,7,7,8,8,9,9,10,10,10a-Nonadecafluorodecahydropyrido[1,2-a]azepine	95827-25-1	−8.7

**Table 5:** Top-Ranked PFAS Ligands Docked at the PXR Orthosteric Site.

DTXSID	CASRN	IUPAC Name	Binding Affinity (Kcal/Mol)
DTXSID501041016	548470-06-0	Cyclohexanecarboxamide, N,N′-[4-(phenylazo)-1,3-phenylene]bis[1,2,2,3,3,4,4,5,5,6,6-undecafluoro- (9CI)	−13.5
DTXSID401026885	NOCAS_1026885	4-Fluoro-N-[1-(4-fluorophenyl)-1-hydroxy-3-(2,2,3,3-tetrafluoro-1,4-benzodioxin-6-yl)propan-2-yl]naphthalene-1-carboxamide	−13.1
DTXSID60881337	125061-94-1	Perfluoro(4a-(cyclohexylmethyl)decahydronaphthalene)	−12.6
DTXSID701026989	NOCAS_1026989	1-[3-Fluoro-1-methyl-2-oxo-3-(trifluoromethyl)indol-5-yl]-3-[[2-methyl-3-(trifluoromethyl)phenyl]methyl]-2,4-dioxopyrimidine-5-carboxylic acid	−12.4
DTXSID70896735	146304-71-4	3,3′-(1,4-Phenylene)bis[5-(tridecafluorohexyl)-1,2,4-oxadiazole]	−12.1
DTXSID10896198	446043-85-2	1~5~,4~5~-Bis(tridecafluorohexyl)-1~2~,2~2~:2~5~,3~2~:3~5~,4~2~-quaterthiophene	−12.1
DTXSID90984683	662-28-2	Hexacosafluorohexadecahydrofluoranthene	−12
DTXSID70597457	51344-02-6	Hexatriacontafluorotetracosahydrocoronene	−12
DTXSID801026875	NOCAS_1026875	4-[2-[4-[3,3,4,4,5,5-Hexafluoro-2-[6-[2-(4-methoxyphenyl)ethenyl]-2-methyl-1-benzothiophen-3-yl]cyclopenten-1-yl]-3,5-dimethylthiophen-2-yl]ethenyl]benzonitrile	−12
DTXSID60896264	919489-99-9	3-(Pentadecafluoroheptyl)-5-(pentafluorophenyl)-1,2,4-oxadiazole	−12
DTXSID40881335	116265-66-8	Perfluoroperhydrobenzyl tetralin	−12

## Data Availability

Data is contained within the article or Supplementary Material.

## Supplementary Materials

The following supporting information can be downloaded at: https://www.mdpi.com/article/10.3390/ijms25158241/s1.
