# Supplementary material for: Large-Scale Screening of Per- and Polyfluoroalkyl Substance Binding Interactions and Their Mixtures with Nuclear Receptors
Source: Int J Mol Sci. 2024 Jul 28;25(15):8241. doi: 10.3390/ijms25158241 (PMC11312074; doi:10.3390/ijms25158241)
Supplement: Supplementary file 1 [file ijms-25-08241-s001.zip › Supplementary Figures.pdf]

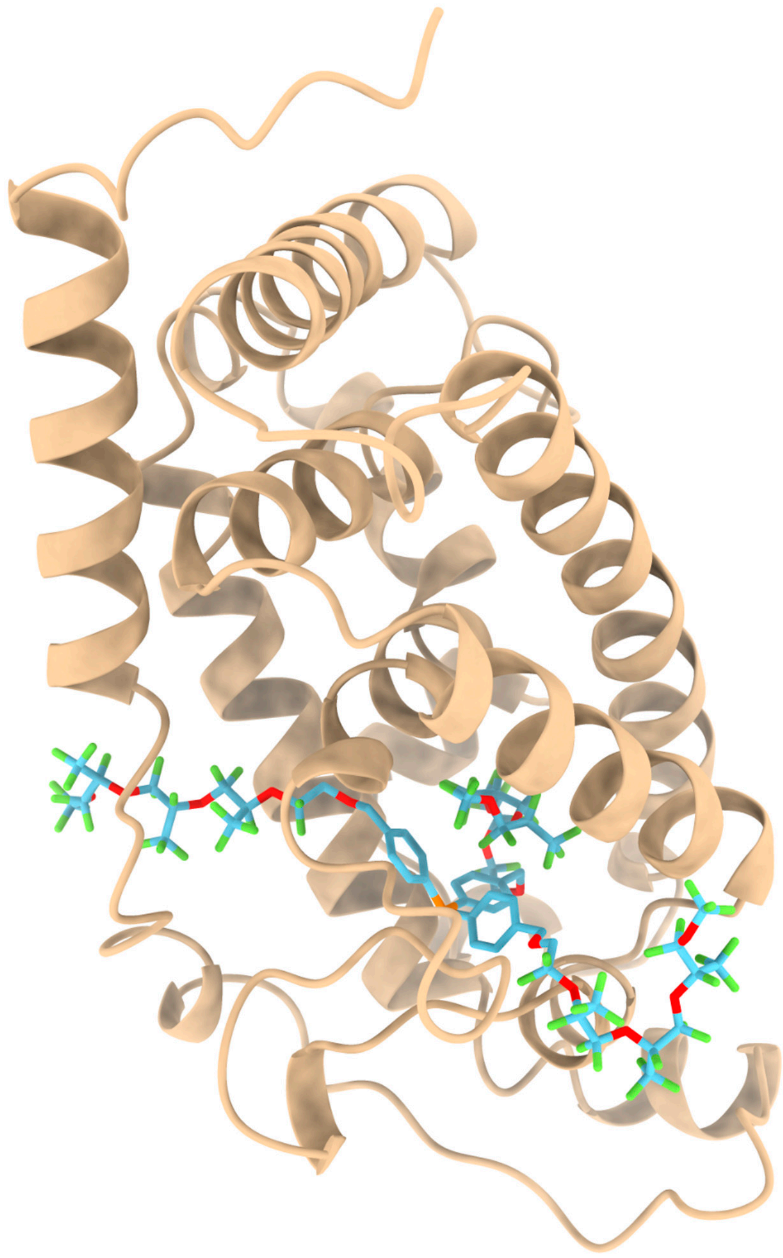

Supplementary Figure 1: DTXSID101034031 is displayed at the VDR orthosteric site. Due to the size of the PFAS, it cannot properly bind to the VDR orthosteric site and stay within the receptor. Due to the inability to bind, DTXSID101034031 is a positive binder at +2.5 kcal/mol.

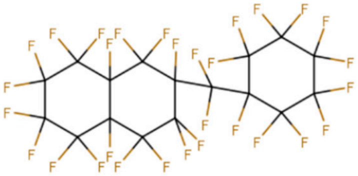

Perfluoroperhydrobenzyl tetralin
Docking Score: -14.7
DTXSID40881335

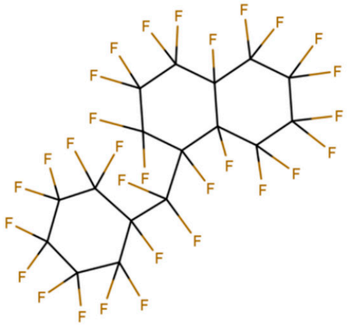

Perfluoro(4a-(cyclohexylmethyl)decahydronaphthalene)
Docking Score: -14.5
DTXSID60881337

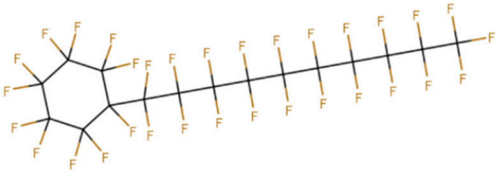

Cyclohexane, 1,1,2,2,3,3,4,4,5,5,6-undecafluoro-6-(1,1,2,2,3,3,4,4,5,5,6,6,7,7,8,8,9,9,10,10,10-heneicosafluorodecyl)-
Docking Score: -13.6
DTXSID101023399

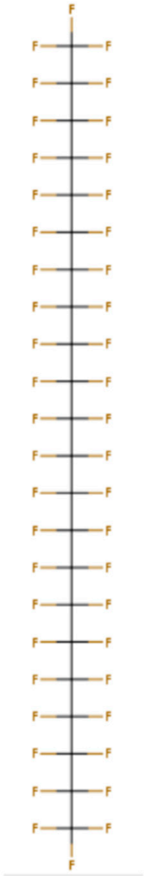

Hexatetracontafluorodocosane
Docking Score: -13.4
DTXSID60895499

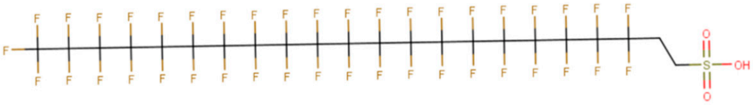

2-(Perfluoroeicosanyl)ethane-1-sulfonic acid
Docking Score: -13.4
DTXSID701036930

Supplementary Figure 2: The top 5 PFAS chemical structure docked into the orthosteric site of the VDR and their respective docking scores are displayed.

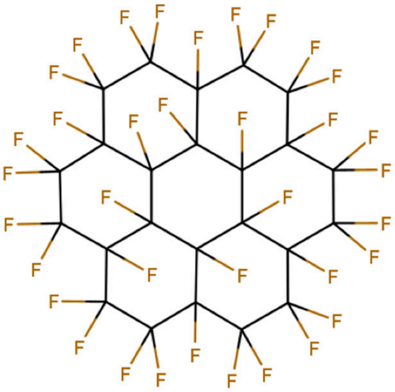

Hexatriacontafluorotetracosahydrocoronene
Docking Score: -10.4
DTXSID70597457

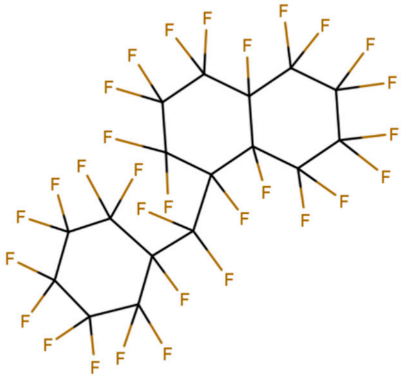

Perfluoro(4a-(cyclohexylmethyl)decahydronaphthalene)
Docking Score: -9.9
DTXSID60881337

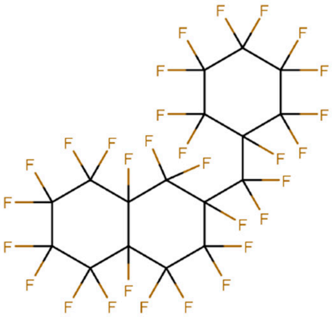

Perfluoroperhydrobenzyl tetralin
Docking Score: -9.8
DTXSID40881335

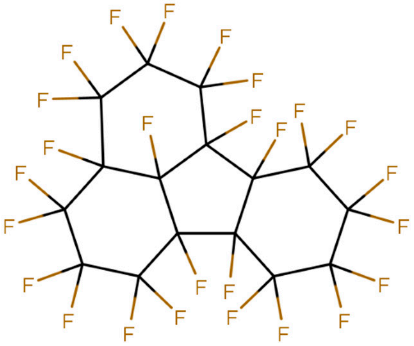

Hexacosafluorohexadecahydrofluoranthene
Docking Score: -9.8
DTXSID90984683

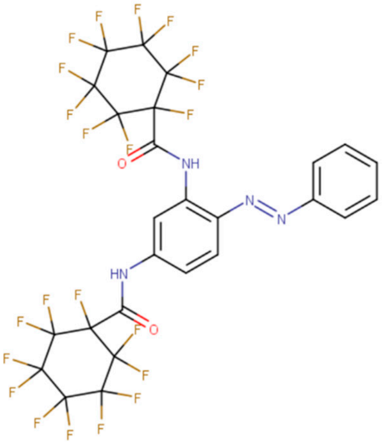

Cyclohexanecarboxamide, N,N'-[4-(phenylazo)-1,3-phenylene]bis[1,2,2,3,3,4,4,5,5,6,6-undecafluoro- (9CI)
Docking Score: -9.6
DTXSID501041016

Supplementary Figure 3: The top 5 PFAS chemical structures docked into the allosteric site of the VDR and their respective docking scores are displayed.

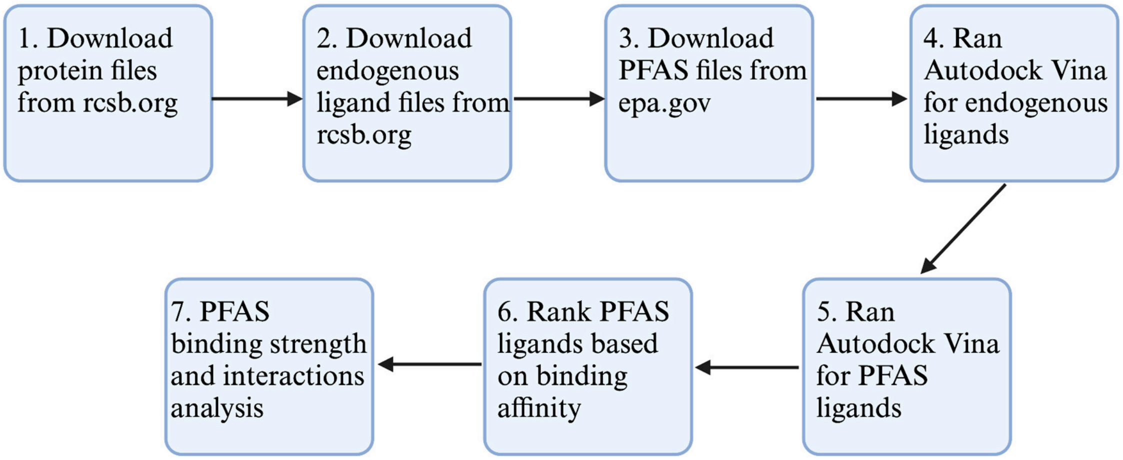

Supplementary Figure 4: Our docking process analyzes the binding interactions between receptor and ligands to understand how different PFAS chemicals can compete with the nuclear receptors at their orthosteric and allosteric sites that cause dysregulation to nuclear receptors. We first retrieved all the required protein files from the rcsb.org protein data bank; two files per protein, one for allosteric and the other for orthosteric sites. Along with the protein files, we obtained the endogenous ligand files from the same protein data bank website. Next, we retrieved all the PFAS files from the Environmental Protection Agency (EPA) CompTox chemical dashboard (https://www.epa.gov/comptox-tools/comptox-chemicals-dashboard). We ran Autodock Vina on the endogenous ligands with the corresponding protein files, and placed both files in UCSF ChimeraX to visualize the superimposition, with an RMSD value less than or equal to 2Å. Once Autodock vina proved to have an accurate docking for the endogenous ligands, we performed the same docking procedure for the PFAS chemical screening, using the python code (Supplementary file Python Code 2). The output is a large array of PFAS, ranked by their binding affinity in kcal/mol, with their proper conformation with respect to the specific nuclear receptors' binding sites. Once we had the ranked PFAS at the respective binding sites for NR proteins, we analyzed the high energy binding PFAS structures to understand which PFAS will have higher likelihood of competing with the NRs activity. Finally, we developed several PFAS mixed model scenarios where we displayed combinations of PFAS and endogenous ligands that can bind and interfere with NRs activity and induce dysregulatory effects.
