# Supplementary material for: Large-Scale Screening of Per- and Polyfluoroalkyl Substance Binding Interactions and Their Mixtures with Nuclear Receptors
Source: Int J Mol Sci. 2024 Jul 28;25(15):8241. doi: 10.3390/ijms25158241 (PMC11312074; doi:10.3390/ijms25158241)
Supplement: Supplementary file 1 [file ijms-25-08241-s001.zip › Supplementary file Python Code.pdf]

Supplementary Python Code 1

```
folder_path = 'C:/Windows/gypsum_dl-1.2.1/updated' # Replace with the actual folder path
for filename in os.listdir(folder_path):
if filename.endswith('.sdf'):
input_file = os.path.join(folder_path, filename)
output_file = os.path.splitext(filename)[0] + '.pdbqt'
command = f'obminimize -o pdbqt -sd -ff GAFF -h {input_file} > {output_file}'
os.system(command)
import os
```

Supplementary Pyhton Code 2

```
import os

x = (r'C:\Users\James\OneDrive\Desktop\PFAS')

for y in os.listdir(x):
    if y.endswith('.pdbqt'):
        startfile = os.path.join(x, y)
        endfile = os.path.splitext(y)[0] + '.txt'
        command = f' vina.exe --ligand {startfile} --config config.txt --log {endfile} '
        os.system(command)
```
